# Controlling *AOX1* promoter strength in *Pichia pastoris* by manipulating poly (dA:dT) tracts

**DOI:** 10.1038/s41598-018-19831-y

**Published:** 2018-01-23

**Authors:** Jun Yang, Haiming Cai, Jie Liu, Min Zeng, Jiawei Chen, Qingmei Cheng, Linghua Zhang

**Affiliations:** Guangdong Provincial Key Laboratory of Protein Function and Regulation in Agricultural Organisms, College of Life Sciences, South China Agricultural University, Guangzhou, Guangdong, 510642 China

## Abstract

Alcohol oxidase I (*AOX1*) promoter is the most popular but strictly-regulated methanol inducible promoter for heterologous protein expression in *Pichia pastoris*. In recent years, *AOX1* promoter libraries have been developed with deletion or insertion methods. The present research manipulated poly (dA:dT) tracts in this promoter to control promoter strength, which hadn’t been tried before. There were 34 variants derived from the native *AOX1* promoter constructed. And variants were integrated into the same genomic location and upstream of the same reporter gene porcine growth hormone (*pGH*). To test the transferability of the results obtained from reporter gene *pGH*, the variants were connected to reporter gene *Lac Z*. The resulted promoter library spanned an activity range between 0.25 and 3.5 fold of the wild-type promoter activity. In addition, activities of variants correlated with their predicted nucleosome architecture, which were directed by poly (dA:dT) tracts. The cumulative sum of predicted nucleosome affinity across the region (−820 to −540) was related to promoters strength in single deletion variants on a proportional basis. Overall, the research promotes understanding of the regulatory patterns for *AOX1* promoter and suggested that varying promoter expression of engineering nucleosome architecture was also a feasible approach in *P*. *pastoris*.

## Introduction

The methylotrophic yeast *Pichia pastoris* (*Komagataella phaffi*) has been commonly used for production of heterologous protein^1,2^. Alcohol oxidase I (*AOX1*) promoter, remarkably strong and tightly regulated by methanol^3,4^, turns out to be most popular. Though with favorable properties and industrial importance,* AOX1* promoter lacks further study, considering the fact that its transcriptional regulatory mechanism has not been clarified^3^. Fine-tuning of heterologous gene expression is necessary for maximization of protein expression level. And developing an efficient strategy for optimizing promoter activity should be based on a better understanding of regulatory mechanism of promoter.

Previous studies of optimizing *AOX1* promoter have focused on deletion and duplication of putative transcription factor-binding sites and identification of upstream activation sequence^5,6^. Although those traditional methods have proved successful in creating promoter library, the resulted variants activities were ~1.6 fold of wild-type *AOX1* promoter activity^5^. There have been great efforts made to study rational design of *AOX1* core promoter in recent years. For instance, Thomas Vogl *et al*.^3^ designed AOX1 core promoter by using a consensus sequence of natural core promoters and common transcription factor binding site motifs. And the activities of synthetic variants ranged from 10% to 117% of wild-type *AOX1* promoter. Besides, Portela RM *et al*.^4^ designed 112 synthetic promoters based on sequence/function relationship of natural core promoters, nucleosome occupancy and the presence of short motifs and fused synthetic core promoters to *AOX1* *cis*-regulatory modules (CRMs). These studies provided new idea for engineering promoters including *AOX1* promoter.

Poly (dA:dT) tracts-homopolymeric stretches of deoxyadenosine are highly abundant in yeast genome^7,8^. These sequences harbor a shorter helical structure and a narrow minor groove, which serves to resist the bending required for histone binding^9^. Poly (dA:dT) tracts could create a “barrier” that favors the formation of highly positioned nucleosomes adjacent to these tracts, which in turn directs the positions of neighboring nucleosomes^10^. In this sense, these tracts could exert influence on nucleosome occupancy and affinity. Many studies show that poly (dA:dT) tracts are important for transcriptional regulation^11,12^. Due to their unusual structure, nucleosomes are strongly depleted from these tracts and their own flanking sequence. Moreover, narrowing nucleosome occupancy over the region in vicinity of those tracts can increase the accessibility of DNA in the same region to transcriptional factor binding sites^7^. Poly (dA:dT) tracts have been used to regulate promoters in yeast. Altering the presence and length of native poly (dA:dT) tracts can increase accessibility to the nearby transcription factor-binding sites which are covered by nucleosomes, thus regulating promoter activities^13,14^.

Poly (dA:dT) tracts are highly prevalent in yeast promoters including *AOX1* promoter. Therefore, the present research aims to analyze the transcriptional effect of poly (dA:dT) tracts in *AOX1* promoter by altering the presence and length of native poly (dA:dT) element in different sites. This study indicates that deletion or lengthening native poly (dA:dT) tracts can alter the variants activities ranging from ~0.25 to ~3.5 fold of wild-type promoter activity, proving that the method is more effective than traditional methods for improving *AOX1* promoter activities. In addition, there is also prediction on occupancy and affinity of nucleosomes. And research shows that occupancy and affinity of nucleosomes in certain regions of *AOX1* promoter are correlated with promoter activities.

## Results

### Designing *AOX1* Promoter Library by Deleting/Adding Poly (dA:dT) Tracts

**Figure 1:**
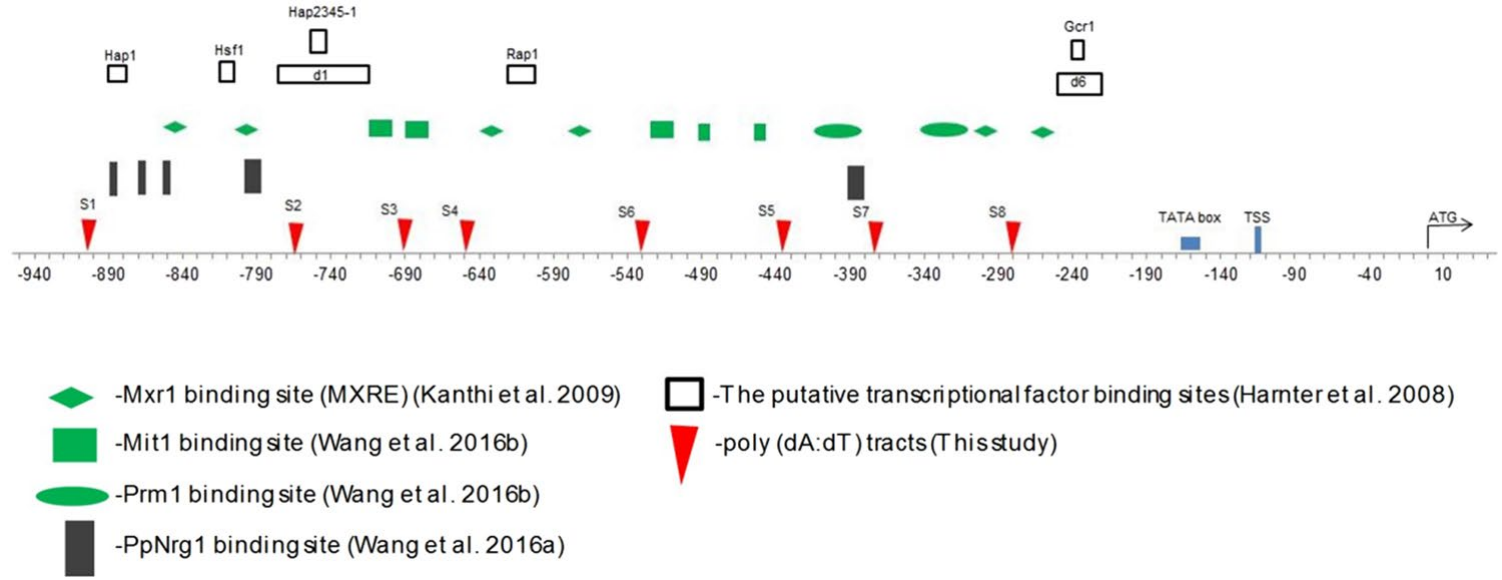
*Cis*-acting sequence elements of the *AOX1* promoter. A schematic representation of *AOX1* promoter sequence was offered. *Cis*-acting elements for regulators containing activators: Mxr1, methanol-induced transcription factor1 (Mit1) and Prm1, repressor: PpNrg1 and putative TFBS identified by Hartner *et al*. were offered. Several poly (dA:dT) tracts in this study: S1: TTTTT (−903 to −899); S2: AAAAA (−767 to −763); S3: TTTATTA (−690 to −684); S4: TTTGTTTATTT (−648 to −638); S5: AAAAAGAAA (−435 to −427); S6: TTTAAA (−530 to −525); S7: AAAAATAAT (−372 to −364) and S8: TTTTT (−281 to −277) were shown.

There are a considerable number of poly (dA:dT) tracts in eukaryotic genome, especially in promoters. Manipulation of poly (dA:dT) tracts can cause changes of nucleosome organization, thus altering the promoter strength^14^. In order to study the influence of poly (dA:dT) on transcription, there were 34 variants constructed on the basis of the native *Pichia pastoris AOX1* promoter. Eight perfect poly (dA:dT) tracts and imperfect tracts (Fig. 1) were identified and used for altering the presence and length of a native poly(dA:dT) element. Deleting identified poly (dA:dT) tracts or adding perfect poly(dA:dT) tracts in these poly(dA:dT) sites respectively can alter the presence and length of a native poly(dA:dT) element.

To measure the promoter activity of these variants, the promoters were connected to reporter gene, porcine growth hormone (pGH) and Lac Z respectively (Table 1). The resulted expression cassettes were integrated into the same genomic location (*GAP* promoter locus) in all strains. The copy numbers of reporter gene, *pGH* and *Lac Z* in transformants were confirmed by qPCR (Table 1). Finally, methods including qPCR, westernbloting (pGH) and beta-galactosidase activity (Lac Z) were adopted to measure the strength of promoter variants.

**Table 1:** The plasmids and corresponding clones used in this study. The copy number of reporter gene in strains were obtained by means of qPCR and are represented by the mean ± SD of three independent experiments.

Plasmids	Corresponding clones	Reporter gene copies	Mutation sites in *AOX1* promoter
pPICZA	—	—	
pGAPZαA	—	—	
pPICZA-pGH	—	—	
pPICZA-pGH-Xba	—	—	
pPICZA-LacZ	—	—	
GAP-pPICZA-pGH	WT	1.35 ± 0.19	
GAP-pPICZA-Del-S1-pGH	Del-S1	1.55 ± 0.21	Deletion of S1 tract within *P*_*AOX1*_
GAP-pPICZA-Del-S2-pGH	Del-S2	0.89 ± 0.31	Deletion of S2 tract within *P*_*AOX1*_
GAP-pPICZA-Del-S3-pGH	Del-S3	1.97 ± 0.62	Deletion of S3 tract within *P*_*AOX1*_
GAP-pPICZA-Del-S4-pGH	Del-S4	1.21 ± 0.6	Deletion of S4 tract within *P*_*AOX1*_
GAP-pPICZA-Del-S5-pGH	Del-S5	1.03 ± 0.06	Deletion of S5 tract within *P*_*AOX1*_
GAP-pPICZA-Del-S6-pGH	Del-S6	1.45 ± 0.36	Deletion of S6 tract within *P*_*AOX1*_
GAP-pPICZA-Del-S7-pGH	Del-S7	0.71 ± 0.18	Deletion of S7 tract within *P*_*AOX1*_
GAP-pPICZA-Del-S8-pGH	Del-S8	0.92 ± 0.25	Deletion of S8 tract within *P*_*AOX1*_
GAP-pPICZA-Add-S1-pGH	Add-S1	1.02 ± 0.28	Addition of 15 bp poly dA:dT tracts in S1 site
GAP-pPICZA-Add-S2-pGH	Add-S2	1.18 ± 0.21	Addition of 15 bp poly dA:dT tracts in S2 site
GAP-pPICZA-Add-S3-pGH	Add-S3	1.02 ± 0.08	Addition of 15 bp poly dA:dT tracts in S3 site
GAP-pPICZA-Add-S4-pGH	Add-S4	1.21 ± 0.11	Addition of 15 bp poly dA:dT tracts in S4 site
GAP-pPICZA-Add-S5-pGH	Add-S5	1.47 ± 0.16	Addition of 15 bp poly dA:dT tracts in S5 site
GAP-pPICZA-Add-S6-pGH	Add-S6	1.14 ± 0.06	Addition of 15 bp poly dA:dT tracts in S6 site
GAP-pPICZA-Add-S7-pGH	Add-S7	1.05 ± 0.02	Addition of 15 bp poly dA:dT tracts in S7 site
GAP-pPICZA-Add-S8-pGH	Add-S8	1.02 ± 0.24	Addition of 15 bp poly dA:dT tracts in S8 site
GAP-pPICZA-Double-Del-S12-pGH	Double-Del-S12	1.04 ± 0.16	Deletion of S1 and S2 tracts within *P*_*AOX1*_
GAP-pPICZA-Double-Del-S24-pGH	Double-Del-S24	0.97 ± 0.20	Deletion of S2 and S4 tracts within *P*_*AOX1*_
GAP-pPICZA-Double-Del-S34-pGH	Double-Del-S34	1.29 ± 0.30	Deletion of S3and S4 tracts within *P*_*AOX1*_
GAP-pPICZA-Double-Del-S46-pGH	Double-Del-S46	1.05 ± 0.31	Deletion of S4and S6 tracts within *P*_*AOX1*_
GAP-pPICZA-Double-Del-S56-pGH	Double-Del-S56	0.86 ± 0.21	Deletion of S5 and S6 tracts within *P*_*AOX1*_
GAP-pPICZA-Double-Del-S57-pGH	Double-Del-S57	1.38 ± 0.06	Deletion of S5 and S7 tracts within *P*_*AOX1*_
GAP-pPICZA-Double-Del-S58-pGH	Double-Del-S58	0.83 ± 0.40	Deletion of S5 and S8 tracts within *P*_*AOX1*_
GAP-pPICZA-Double-Del-S67-pGH	Double-Del-S67	1.27 ± 0.47	Deletion of S6 and S7 tracts within *P*_*AOX1*_
GAP-pPICZA-Double-Del-S78-pGH	Double-Del-S78	0.80 ± 0.03	Deletion of S7 and S8 tracts within *P*_*AOX1*_
GAP-pPICZA-Double-Add-S12-pGH	Double-Add-S12	1.27 ± 0.23	Addition of 15 bp poly dA:dT tract in S1 and S2 tracts within *P*_*AOX1*_
GAP-pPICZA-Double-Add-S24-pGH	Double-Add-S24	1.27 ± 0.12	Addition of 15 bp poly dA:dT tract in S2 and S4 tracts within *P*_*AOX1*_
GAP-pPICZA-Double-Add-S34-pGH	Double-Add-S34	0.84 ± 0.09	Addition of 15 bp poly dA:dT tract in S3 and S4 tracts within *P*_*AOX1*_
GAP-pPICZA-Double-Add-S46-pGH	Double-Add-S46	1.01 ± 0.17	Addition of 15 bp poly dA:dT tract in S4 and S6 tracts within *P*_*AOX1*_
GAP-pPICZA-Double-Add-S56-pGH	Double-Add-S56	0.92 ± 0.19	Addition of 15 bp poly dA:dT tract in S5 and S6 tracts within *P*_*AOX1*_
GAP-pPICZA-Double-Add-S57-pGH	Double-Add-S57	1.15 ± 0.30	Addition of 15 bp poly dA:dT tract in S5 and S7 tracts within *P*_*AOX1*_
GAP-pPICZA-Double-Add-S58-pGH	Double-Add-S58	1.28 ± 0.27	Addition of 15 bp poly dA:dT tract in S5 and S8 tracts within *P*_*AOX1*_
GAP-pPICZA-Double-Add-S67-pGH	Double-Add-S67	1.13 ± 0.12	Addition of 15 bp poly dA:dT tract in S6 and S7 tracts within *P*_*AOX1*_
GAP-pPICZA-Double-Add-S78-pGH	Double-Add-S78	1.38 ± 0.45	Addition of 15 bp poly dA:dT tract in S7 and S8 tracts within *P*_*AOX1*_
GAP-pPICZA-LacZ	WT-L	1.17 ± 0.05	
GAP-pPICZA-Del-S1-LacZ	Del-S1-L	1.04 ± 0.25	Deletion of S1 tract within *P*_*AOX1*_
GAP-pPICZA-Del-S2-LacZ	Del-S2-L	1.19 ± 0.3	Deletion of S2 tract within *P*_*AOX1*_
GAP-pPICZA-Del-S3-LacZ	Del-S3-L	2.07 ± 0.58	Deletion of S3 tract within *P*_*AOX1*_
GAP-pPICZA-Del-S4-LacZ	Del-S4-L	1.19 ± 0.20	Deletion of S4 tract within *P*_*AOX1*_
GAP-pPICZA-Del-S5-LacZ	Del-S5-L	1.37 ± 0.21	Deletion of S5 tract within *P*_*AOX1*_
GAP-pPICZA-Del-S6-LacZ	Del-S6-L	1.11 ± 0.23	Deletion of S6 tract within *P*_*AOX1*_
GAP-pPICZA-Del-S7-LacZ	Del-S7-L	1.12 ± 0.02	Deletion of S7 tract within *P*_*AOX1*_
GAP-pPICZA-Del-S8-LacZ	Del-S8-L	0.86 ± 0.23	Deletion of S8 tract within *P*_*AOX1*_
GAP-pPICZA-Add-S1-LacZ	Add-S1-L	0.77 ± 0.01	Addition of 15 bp poly dA:dT tracts in S1 site
GAP-pPICZA-Add-S2-LacZ	Add-S2-L	0.79 ± 0.30	Addition of 15 bp poly dA:dT tracts in S2 site
GAP-pPICZA-Add-S3-LacZ	Add-S3-L	1.02 ± 0.10	Addition of 15 bp poly dA:dT tracts in S3 site
GAP-pPICZA-Add-S4-LacZ	Add-S4-L	1.14 ± 0.31	Addition of 15 bp poly dA:dT tracts in S4 site
GAP-pPICZA-Add-S5-LacZ	Add-S5-L	0.86 ± 0.01	Addition of 15 bp poly dA:dT tracts in S5 site
GAP-pPICZA-Add-S6-LacZ	Add-S6-L	1.73 ± 0.28	Addition of 15 bp poly dA:dT tracts in S6 site
GAP-pPICZA-Add-S7-LacZ	Add-S7-L	0.88 ± 0.01	Addition of 15 bp poly dA:dT tracts in S7 site
GAP-pPICZA-Add-S8-LacZ	Add-S8-L	0.88 ± 0.21	Addition of 15 bp poly dA:dT tracts in S8 site
GAP-pPICZA-Double-Del-S12-LacZ	Double-Del-S12-L	1.15 ± 0.26	Deletion of S1 and S2 tracts within *P*_*AOX1*_
GAP-pPICZA-Double-Del-S24-LacZ	Double-Del-S24-L	1.50 ± 0.02	Deletion of S2 and S4 tracts within *P*_*AOX1*_
GAP-pPICZA-Double-Del-S34-LacZ	Double-Del-S34-L	1.30 ± 0.37	Deletion of S3and S4 tracts within *P*_*AOX1*_
GAP-pPICZA-Double-Del-S46-LacZ	Double-Del-S46-L	1.10 ± 0.16	Deletion of S4and S6 tracts within *P*_*AOX1*_
GAP-pPICZA-Double-Del-S56-LacZ	Double-Del-S56-L	1.06 ± 0.19	Deletion of S5 and S6 tracts within *P*_*AOX1*_
GAP-pPICZA-Double-Del-S57-LacZ	Double-Del-S57-L	1.27 ± 0.12	Deletion of S5 and S7 tracts within *P*_*AOX1*_
GAP-pPICZA-Double-Del-S58-LacZ	Double-Del-S58-L	0.98 ± 0.06	Deletion of S5 and S8 tracts within *P*_*AOX1*_
GAP-pPICZA-Double-Del-S67-LacZ	Double-Del-S67-L	1.19 ± 0.25	Deletion of S6 and S7 tracts within *P*_*AOX1*_
GAP-pPICZA-Double-Del-S78-LacZ	Double-Del-S78-L	1.50 ± 0.34	Deletion of S7 and S8 tracts within *P*_*AOX1*_
GAP-pPICZA-Double-Add-S12-LacZ	Double-Add-S12-L	1.05 ± 0.01	Addition of 15 bp poly dA:dT tract in S1 and S2 tracts within *P*_*AOX1*_
GAP-pPICZA-Double-Add-S24-LacZ	Double-Add-S24-L	1.01 ± 0.28	Addition of 15 bp poly dA:dT tract in S2 and S4 tracts within *P*_*AOX1*_
GAP-pPICZA-Double-Add-S34-LacZ	Double-Add-S34-L	0.90 ± 0.27	Addition of 15 bp poly dA:dT tract in S3 and S4 tracts within *P*_*AOX1*_
GAP-pPICZA-Double-Add-S46-LacZ	Double-Add-S46-L	0.87 ± 0.19	Addition of 15 bp poly dA:dT tract in S4 and S6 tracts within *P*_*AOX1*_
GAP-pPICZA-Double-Add-S56-LacZ	Double-Add-S56-L	0.94 ± 0.23	Addition of 15 bp poly dA:dT tract in S5 and S6 tracts within *P*_*AOX1*_
GAP-pPICZA-Double-Add-S57-LacZ	Double-Add-S57-L	1.29 ± 0.15	Addition of 15 bp poly dA:dT tract in S5 and S7 tracts within *P*_*AOX1*_
GAP-pPICZA-Double-Add-S58-LacZ	Double-Add-S58-L	0.83 ± 0.07	Addition of 15 bp poly dA:dT tract in S5 and S8 tracts within *P*_*AOX1*_
GAP-pPICZA-Double-Add-S67-LacZ	Double-Add-S67-L	0.86 ± 0.17	Addition of 15 bp poly dA:dT tract in S6 and S7 tracts within *P*_*AOX1*_
GAP-pPICZA-Double-Add-S78-LacZ	Double-Add-S78-L	0.86 ± 0.20	Addition of 15 bp poly dA:dT tract in S7 and S8 tracts within *P*_*AOX1*_

### Predicting Nucleosome Structure in *AOX1* Promoter Mutants

In order to study correlation between nucleosome structure and poly (dA:dT) tracts, there is prediction of nucleosome occupancy of *AOX1* promoter based on the hidden Markov model, of which practicability was validated by previous work^15^. Providing the fact that the hidden Markov model is usually used for predicting genome nucleosome occupancy, the prediction is able to reflect nucleosome occupancy of *AOX1* promoter in repressed state.

Deletion of poly (dA:dT) tracts of most of variants has slight influence on nucleosome positioning. But there is noticeable increase in the predicted affinity of nucleosomes which cover correspondent poly (dA:dT) tracts (Fig. S1a and b). Deletion of poly (dA:dT) tracts increases predicted affinity of nucleosome nearby poly (dA:dT) tracts. In poly (dA:dT)-tracts-deleted variants, which include Del-S1~Del-S8 except for Del-S4, Double-Del-12 and Double-Del56~Double-Del-78, the nucleosome architecture of promoters show fewer changes than that of wild-type *AOX1* promoter (Fig. S2). However, nucleosomes (−5, −4, −3, and −2) of the Del-S4 variant, the Double-Del-24 variant, the Double-Del-46 variant and the Double-Del-34 variant moved forward to a region about 50 base pairs away from promoters. And the distance between −1 nucleosome and −2 nucleosome is further than that of other poly (dA:dT)-tracts-deleted or wild variants. Obviously, the repositioning of nucleosomes of those variants also changes positions of transcription factor binding sites relative to nucleosomes position (Fig. 1 and Fig. S2).

After addition of 15 bp poly (dA:dT) tracts in different sites, there was a reduction in nucleosome occupancy upon addition of a nearby tract. The nucleosomes (−5, −4) positioning of Add-S1 and Add-S3 variants had been influenced significantly. And the gap between nucleosome (−4) and nucleosome (−3) increased by nearly 90 base pairs, which influences the position of nucleosome (−5) and (−4) (Fig. S2). In the Add-S5 variant, nucleosome (−2) was evicted by inserting poly (dA:dT) tracts in S5 site (Fig. S2). The Add-S6 variant and Add-S7 variant shared a similar nucleosome positioning (Fig. S2) while the predicted affinity of nucleosome nearby the S6 or S7 site decreased obviously (Fig. S1c). Compared with promoters of wild type, the number of nucleosome in the variant Add-S4 and Add-S8 showed few changes. However, the position of nucleosome (−1) in Add-S8 moved 50 base pairs towards TSS and almost covered the TATA box (Fig. 1 and Fig. S2). As for the Add-S4 variant, the gap between nucleosome (−3) and nucleosome (−4) turned wider compared with that of wild promoters (Fig. S2). The nucleosome architecture of variants, which contains double sites addition of poly (dA:dT) tracts, had changed. In Double-Add-24, Double-Add-34, Double-Add-46, Double-Add-56 and Double-Add-57, the nucleosomes were evicted by addition of poly (dA:dT) tracts nearby them (Fig. S2 and Fig. 1). In addition, the nucleosome (−1) of Double-Add-58 and Double-Add-78 moved ~50 bp towards TSS and almost covered the TATA box (Fig. 1 and Fig. S2).

### Effect of poly (dA:dT) tracts on expression of reporter gene

**Figure 2:**
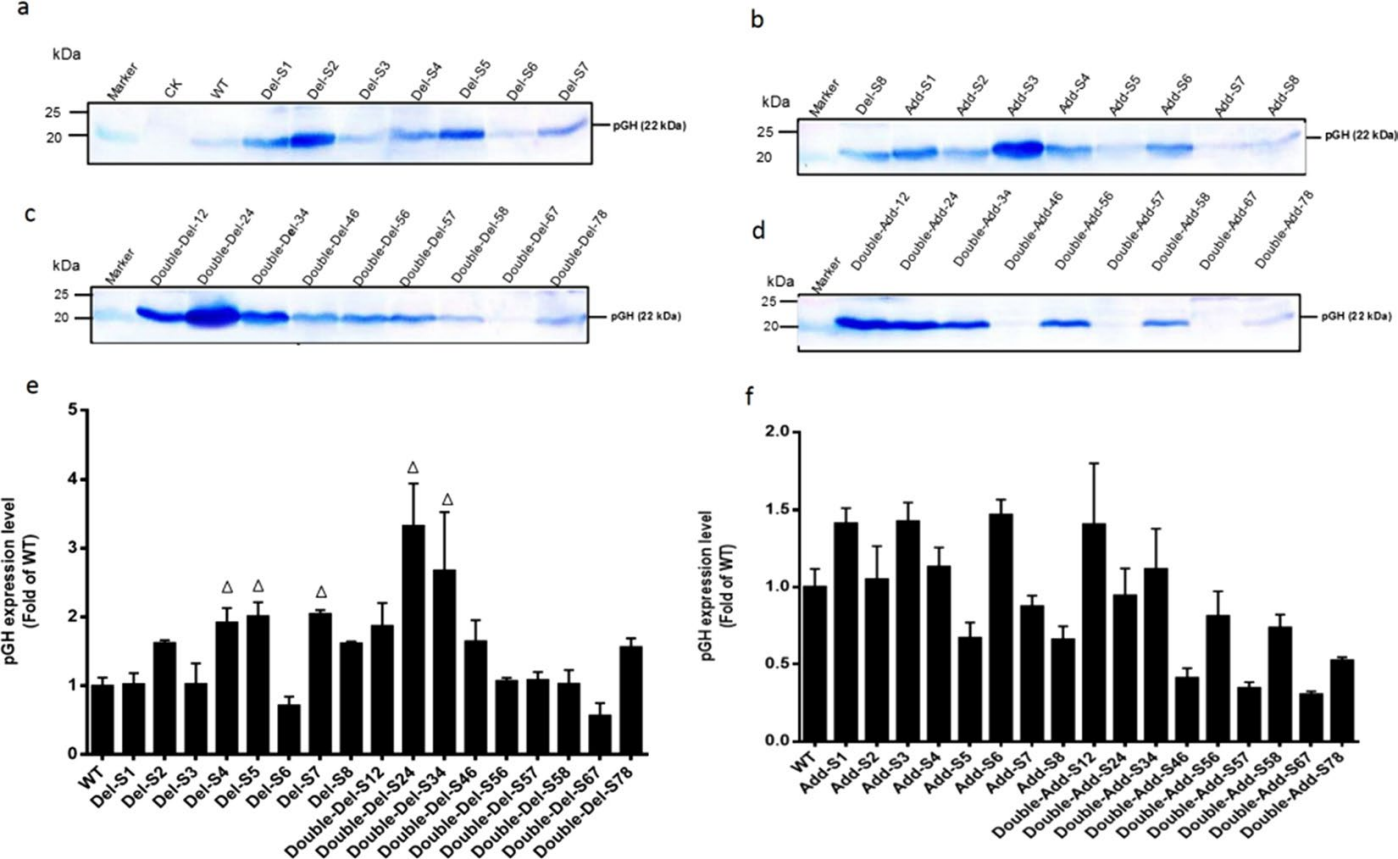
Western blots of pGH protein from strains contained *P*_*AOX1*_ variants. For detection of pGH, 50 μg intracellular proteins were used for SDS-PAGE while Rabbit anti-pGH polyclonal antibody was used for Western blot. The pGH expression level in poly(dA:dT) tracts deletion variants were shown in Lane 3–9 (**a**), Lane 1 (**b**) and Lane 1–9 (**c**); The pGH expression level in addition variants were shown in Lane 2–9 (**b**) and Lane 1–9 (**d**). CK represented negative control samples from pPICZA transformant; WT represented the pGH expression level from strain harboring wild-type *AOX1* promoter. The relative expression levels of pGH in all strains were quantified with ImageJ software. WT was chosen as the standard sample to perform relative comparison. The integrated density of each band represented the abundance of protein. The results of three independent cultivations were indicated as mean ± SD. The relative expression level of pGH in deletion variants were shown in **e** and addition variants in **f**.

**Figure 3:**
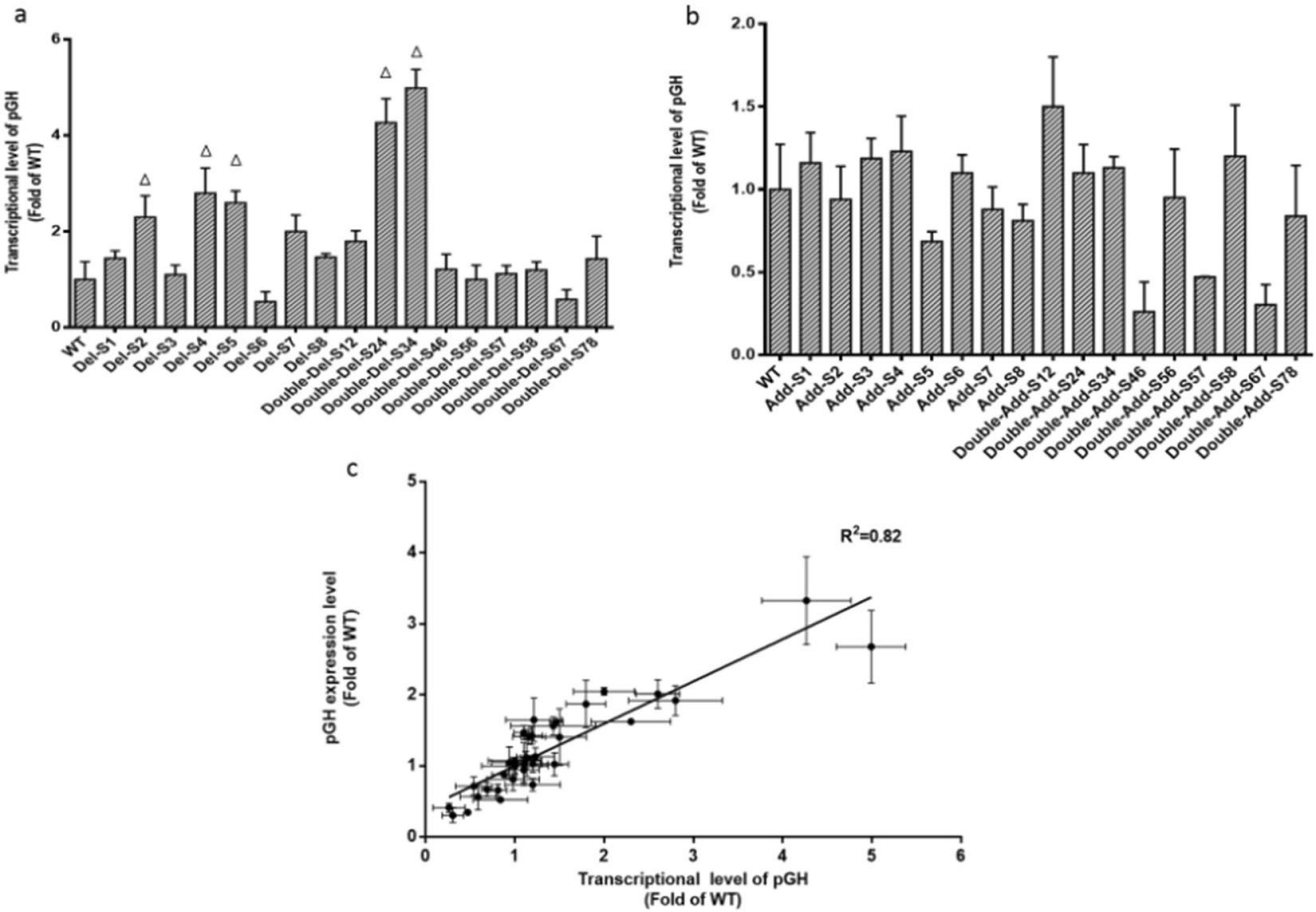
The transcriptional level of reporter gene pGH in strains containing promoter variants and correlation of relative pGH expression level and relative mRNA transcript level. Transcript levels of variants were compared with wild-type promoter transformants, which is represented by the mean ± SD of three independent cultivations. Variants with stronger strength were marked with triangle. The transcriptional level of deletion variants were shown in (**a**) while the transcriptional levels of addition variants were shown in (**b**). (**c)**: pGH expression levels correlated linearly with relative mRNA transcript levels with an R^2^ = 0.82.

Nucleosome architecture plays an important role in tuning yeast promoter activity. And poly (dA:dT) tract is a major factor influencing nucleosome positioning. In order to test the influence of poly dA:dT on *AOX1* promoter activity, there are series of variants containing different length of poly (dA:dT) tracts (Table 1) constructed. And *pGH* is tested by means of western blot and *Lac Z* by beta-galactosidase activity. Firstly, there was deletion of the poly (dA:dT) tract in different sites respectively. Expression levels of *pGH* under variants (Del-S2, S4, S5, S7 and S8) increased to 1.5~2 fold of WT (Fig. 2a,b and e). The variant Del-S6 activity turned lower than WT while the others (Del-S1 and Del-S3) were consistent with WT (Fig. 2a and e). In an attempt to improve the *AOX1* promoter activity, there was deletion of poly (dA:dT) tracts in two sites. As a result, there was a considerable rise in the strength (2~3.5 fold of WT) of variants (Double-Del-12, 24 and 34). However, double deletion in other sites caused no change in the promoter activity (Fig. 2c and e). For the purpose of verifying the influence of poly (dA:dT) tracts on transcription, there was adjustment on the length of the poly (dA:dT) tracts in different sites corresponding to deletion variants, thus measuring the promoter activities. After inserting poly (dA:dT) tracts of 15 bp into those sites in *AOX1* promoter, it was found that activities of the variants (Add-S1, S3, S4 and S6) increased to 1.2~1.5 fold of WT while that of variants (Add-S5, S7 and S8) dropped (Fig. 2b and f). However, except for Double-Add-12, 24 and 34 (Fig. 2d and f), the activities in double deletion sites decreased to 0.25~0.7 fold of WT. In addition, transcription levels of *pGH* in variants were also investigated by means of qPCR (Fig. 3a,b). There was analysis of the relationship between mRNA and protein level, of which results showed that correlation coefficient for variants was 0.82 (Fig. 3c).

**Figure 4:**
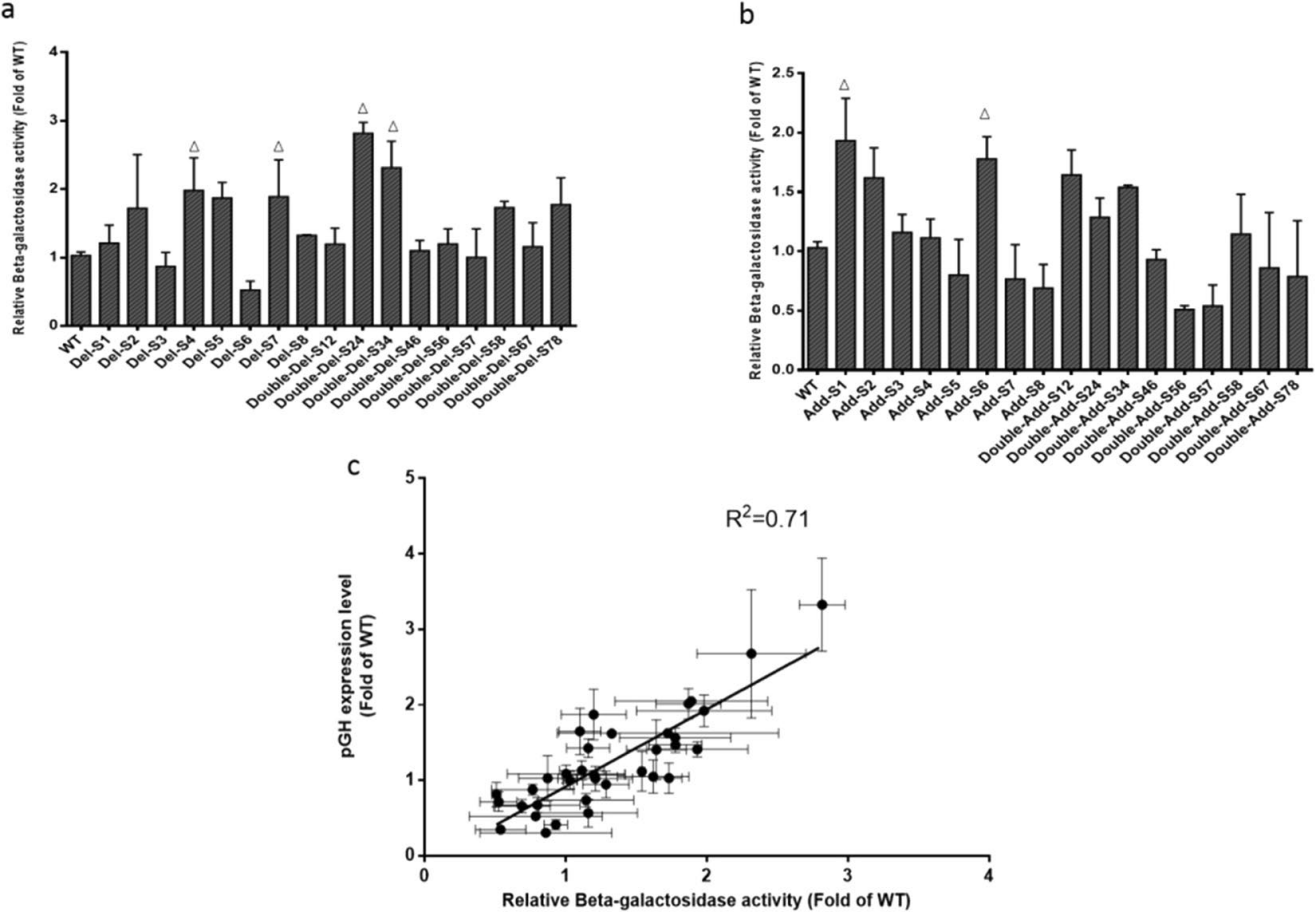
Promoter activity is represented by beta-galactosidase activity. Beta-galactosidase enzyme activities of variants were compared with wild-type promoter transformants, which is represented by the mean ± SD of three independent cultivations. Variants with stronger strength were marked with triangle. Relative enzyme activities of deletion variants were shown in (**a**) while relative enzyme activities of addition variants were shown in (**b**). (**c)** pGH expression levels were linearly related to relative beta-galactosidase activities with an R^2^ = 0.71.

To test the transferability of research results of reporter gene and pGH, the variants were connected to reporter gene and Lac Z. And the transcription levels of beta-galactosidase were also performed (Fig. 4a,b). It is found that the beta-galactosidase activities were in great consistence with the expression level of *pGH* (Fig. 4c). Taken together, results showed that poly (dA:dT) elements could tune the *AOX1* promoter activity. And the promoter activity of poly (dA:dT) –tract-deleted variants were higher than that of promoters with inserting 15 bp poly (dA:dT) tracts.

In order to confirm that *AOX1* expression could strongly restrict glucose/glycerol, the strength of wild-type and variant* AOX1* promoters in the presence of glycerol by beta-galactosidase activities were also measured here. The activities of beta-galactosidase increased to 1.5~2.5 fold of WT apart from variants (Del-S3, S6 and Double-Del-S46, S56) (Fig. S3a). And the activities of beta-galactosidase in the variants (Add-S2, S3, S4 and S6) increased to 1.4~1.5 fold of WT (Fig. S3b). Analysis of the relationship between the activities of beta-galactosidase without induction of methanol and that with induction of methanol showed that correlation coefficient of variants was 0.56, indicating that the presence of glycerol caused slight changes in the regulation of poly dA:dT tracts on promoter strength.

### The activities of variants correlate with predicted nucleosome architecture

**Figure 5:**
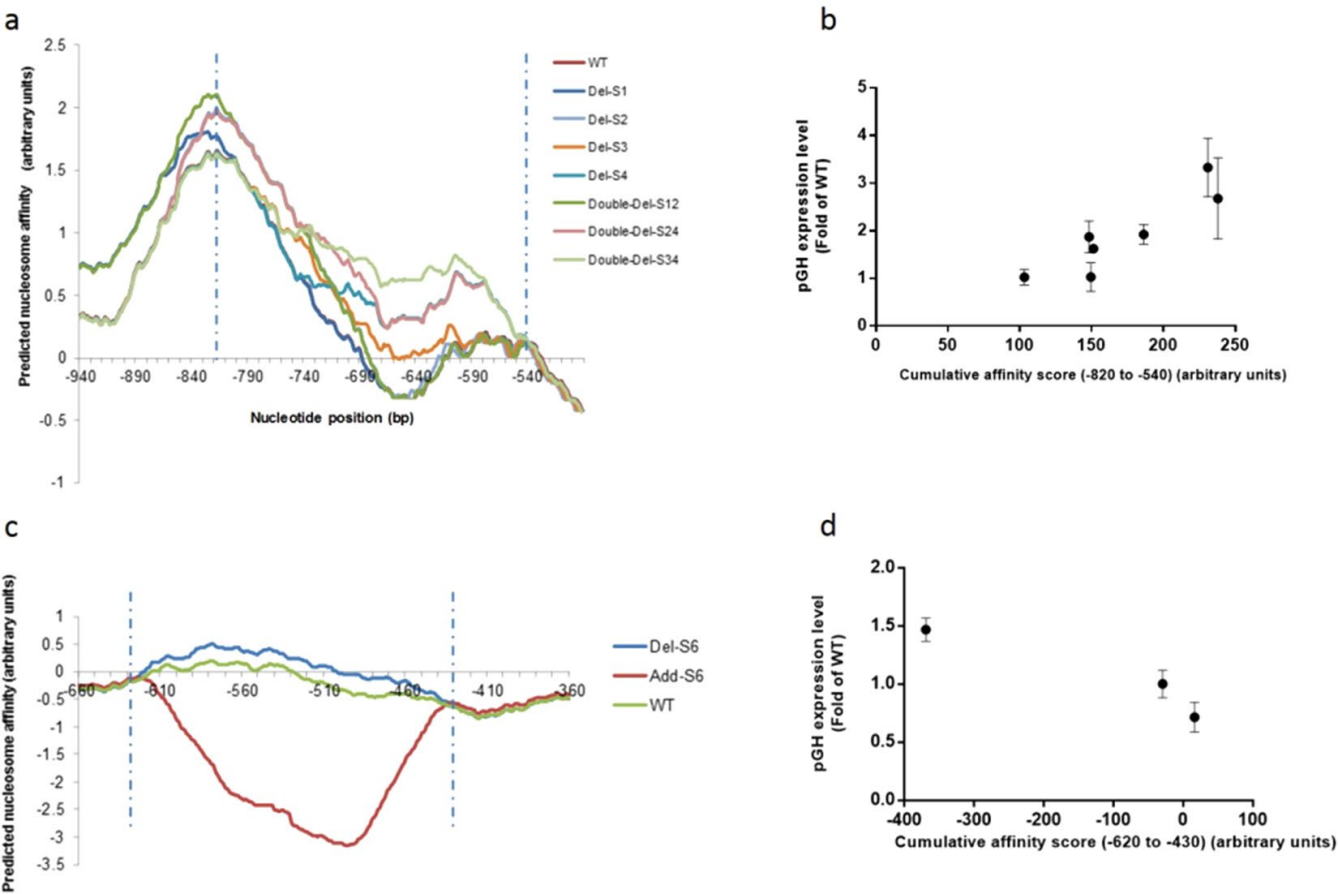
Nucleosome affinity of certain region on *AOX1* promoter is related to mutant promoter strength. Predicted nucleosome affinity profiles of region (−820 to −540) and (−620 to −430), which was generated by NuPoP software for several variants, were shown in (**a**) and (**d**) respectively; the cumulative sum of predicted nucleosome affinity across the region (−820 to 540) was positively related to promoter strength on a proportional basis (**b**); the cumulative sum of predicted nucleosome affinity across the region (−620 to −430) was negatively related to promoter strength on a proportional basis (**d**).

Given that the transcriptional activity could be influenced by changes in poly (dA:dT) tracts, the following part probes into whether influence could be explained through prediction of nucleosome architecture. And the prediction is carried out by manipulating poly (dA:dT) tracts. There was a correlation analysis of relationship between predicted nucleosome architecture and expression level. Nucleosome architectures of Variant Del-S4, Variant Double-Del-S24 and Variant Double-Del-S34 show great similarity. But their nucleosome architectures are significantly different from that of other poly (dA:dT) –tract-deleted variants and WT variants (Fig. S2). Interestingly, three variants exhibited higher transcription level than that of others, which might be attributed to their unusual architectures and relative position of nucleosomes and transcriptional factors binding sites (Figs 1, 2 and Fig. S2). Moreover, according to the profiles of predicted nucleosome affinity of poly (dA:dT) –tract-deleted variants, the predicted nucleosome affinity of some regions in variants was correlated with the corresponding promoter activities. For example, the cumulative sum of predicted nucleosome affinity across the region (−820 to −540) were proportional to promoters strength especially in single poly (dA:dT) –tract-deleted variants (Fig. 5a and b). The nucleosome architecture of poly (dA:dT) –tract-added variants showed noticeable changes comparing with that of WT as well as those adjacent to the insert site, especially in double-poly (dA:dT) –tract-deleted variants (Fig. S2). As shown in Fig. S1a and b, most of double-poly (dA:dT) –tract-deleted variants containing lower nucleosome occupancy showed lower expression level compared with WT (Fig. 2e). Theoretically, there is negligible correlation between predicted nucleosome affinity and promoter activity of poly (dA:dT) –tract-added variants. And according to Fig S1a and b, even predicted nucleosome affinity of extremely low level in regions (from −485 to −255) did not allow for promoter activity.

## Discussion

Alcohol oxidase I (*AOX1*) promoter is the most commonly used and tightly regulated metha2nol inducible promoter for heterologous protein expression in *Pichia pastoris*^4^. In the present research, a *AOX1* promoter library consisting of 34 variants was connected to porcine growth hormone (pGH). And poly (dA:dT) tracts were adjusted, leading to a broad range of activities (from 0.25~3.5 fold of WT). The research indicates that systemic adjustment on poly (dA:dT) tracts in yeast promoters can contribute to create promoters which provide expression levels of a wide range.

Recently, there have been various studies focusing on rational design of *AOX1* core promoter. Thomas Vogl *et al*.^3^ designed *AOX1* core promoter by using a consensus sequence of natural core promoters, common transcription factor binding site motifs and the activities of synthetic variants. Besides, Portela RM *et al*.^4^ designed 112 synthetic promoters based on sequence/function interdependence of natural core promoters, nucleosome occupancy and the presence of short motifs. The researcher also connected synthetic core promoters to *AOX1* cis-regulatory modules (CRMs). However, previous studies, which were based on stochastic series with finite states, failed to figure out factors influencing *AOX1* promoter activity. In the present research, systematic adjustment on poly (dA:dT) tracts is not only suitable for *AOX1* but also useful for other yeast promoters, which can lay foundation for research of engineering promoter other promoters as well as* AOX1* in the future.

Some studies showed that architecture of nucleosome surrounding transcription factor-binding sites could be an important factor influencing the strength of yeast promoters. Nuclesome architecture plays a leading role in defining yeast promoter activity, which makes it able to design synthetic promoters. Poly (dA:dT) tracts not only disturb nucleosome formation but also increase accessibility of transcription factor-binding sites14 nearby, thus promoting expression. Some research indicated that the properties and spatial arrangement of poly (dA:dT) tracts could exert considerable impact on transcription^13,14^. However, the present study provides findings contrary to that of previous research of the influence of poly (dA:dT) tracts in *AOX1* promoter on transcription. Deletion of these tracts in *AOX1* promoter could stimulate expression to some extents while addition of 15 bp poly (dA:dT) tracts in different sites resulted in a reduction in expression level especially in double sites addition variants.

It is speculated that these influences could arise from some specialties of *AOX1* promoter. Many studies focused on the regulatory role of poly (dA:dT) tracts in simple constitutive promoter such as *HIS3* promoter^11,14^. However, there has not any reported research centering on the regulatory role of poly (dA:dT) tracts in inducible promoters including *AOX1* promoter so far. Providing the fact that *AOX1* promoter is regulated by methanol, the regulation by poly (dA:dT) tracts on *AOX1* promoter would be more complex than that on constitutive promoter. According to Fig. 5a and b, increase in predicted affinity of nucleosome in region (−820 to −540) led to the rise in promoter activity. There are a great number of activating transcription binding sites and repressive factor binding sites in the region (Fig. 1). Based on Harnter’s^5^ study, compared to WT, deletion of d1 (−777 to −712) or Rap (−615 to 601) resulted in higher promoter activity, suggesting that this region contains repressive binding site^5^. Despite, with methanol induction, deletion of Hsf1 (−805 to −798) and d6 (−253 to −224) resulted in decreasing promoter activities, the promoter activities in depression (carbon source was depleted) increased, suggesting that these fragments probably served as the binding site for both activators and repressors. Recently, A Cys2His2 zinc finger transcriptional regulator PpNrg1 has been identified as a repressor of *AOX1* promoter^16^. Two binding sites of Cys2His2 zinc finger transcriptional regulator PpNrg1 contain conserved regions with CYCCNY which are binding sites of methanol expression regulator (Mxr1p) while a binding site contains a part of region of Prm1 binding sites^17^. To sum up, there are both of activating binding sites and repressor binding sites distributing on *AOX1* promoters. And some of them even share the same region. These features suggest that competition between activators and repressors plays an important role in regulating transcription of *AOX1* promoter.

PpNrg1 probably competes with activator Mxr1p^18^ on the binding sites of Mxr1p when regulation of *AOX1* promoter works. Increase in predicted nucleosome affinity of these regions can lead to decrease in the binding affinity of repression factor according to the model of Raveh-Sadka’s study^14^. As the promoter is activated by methanol, Mxr1p will translocate to nucleus, thus regulating the promoter. Given that the main competitor for binding is histone, it would be easier for activators to bind on the Mxr1p binding site (MXRE). On the contrary, decrease in the nucleosome affinities of these regions will make Mxr1p compete with PpNrg1which has already been band on the Mxr1p sites before the translocation of Mxr1p into nucleus. This assumption makes it easier to understand the regulatory role of poly (dA:dT) tracts discussed in the study. The regions (−820 to −540) contain PpNrg1 binding sites (−796 to −785) which could also be identified by Mxr1p. Increasing the predicted affinity of nucleosome nearby these Nrg1binding sites could reduce the affinity of Nrg1 repressor. Therefore, compared with Nrg1 which has already been bound to MXRE, Mxr1 can compete with histone in an easier way. In this sense, depressing *AOX1* promoter can improve the efficiency of binding DNA to regulator.

In region (−620 to −430), decreasing predicted nucleosome affinity could improve promoter activity (Fig. 5d). The region contained many activating (e.g. Mxr1p and Mit1) transcription binding sites. The decreasing predicted nucleosome affinity made transcription binding sites more accessible for activators, which resulted in increasing promoter activity. Coincidentally, this region also contains an upstream activation sequence (−638 to −510), which has been verified by previous studies^6^. The activity of mutant *AOX1* promoter, which contains three copies of region (−638 to −510), was 1.57 fold of that of wild-type *AOX1* promoter. With insertion of 15 bp poly dTs in S6 site of *AOX1* promoter, the expression level of pGH increased to ~1.5 fold of wild-type (Fig. 2b and e), proving the function of the upstream activation sequence.

Theoretically, there was negligible correlation between the predicted nucleosome affinity and promoter activity in region (−585 to −185). And the research proved that predicted nucleosome affinity of extremely low level caused slight changes in promoter activity (Fig. S1). Such influence might be attributed to Prm1/PpNrg1 binding site in the region. The activating Prm1 share a same binding region with repressor PpNrg1, as similar with Mxr1p/PpNrg1 binding site in region (−820 to −540).

In addition, reposition of nucleosome in variants has influence in promoter activities. For example, nucleosome (−4) moved forward 50 base pairs to the upstream of promoter in variant Del-S4, variant Double-Del-24, variant Double-Del-46 and variant Double-Del-34, which resulted in location of Mxr1p/PpNrg1 binding sites (Fig. 1) in the middle of −4 nucleosome. In this way, accessibility to PpNrg1 binding site repressor PpNrg1 reduced, leading to improvement in the promoter activity. On one hand, in variant Double-Add-58 and variant Double-Add-78, the nucleosome (−1) moved ~50 bp towards TSS and almost covered the TATA box, which might keep the TATA box inaccessible for the TATA-binding protein (TBP)^19^. So the activities of Double-Add-58 and Double-Add-78 were decreased comparing with wild-type promoter.

On the other hand, the nucleosome architecture of *AOX1* promoter in active state may show great difference from that in repressed state, which also happens to *PHO5* promoter^20^. Both sequence of such promoters and specific remodeled complexes could participate in switching the state of chromatin. The remodeled complexes could invert the nucleosome architecture at a promoter, thus switching the state of chromatin for transcription.

Poly (dA:dT) tracts, which were inserted to regions, may influence the nucleosome repositioning by remodeling complexes when chromatin was in the active state. And poly (dA:dT) tracts, which were extended, may disturb the original positioning of nucleosome when chromatin was in the active state. Therefore, most of double-addition variants showed low level expression in the present study.

There was a promoter library created by deletion and addition of poly (dA:dT) tracts within the *AOX1* promoter sequence in this study. Unprecedentedly, the present research showed that poly (dA:dT) tracts could regulate *AOX1* promoter which is inducible, suggesting that varying promoter expression by engineering nucleosome architecture is also a feasible approach in *P*. *pastoris*. However, there remains a large space for study of the regulation mechanism of poly (dA:dT) tracts in *AOX1* promoter.

## Methods

### Strains and plasmids

Strains: *P*. *pastoris* X33 (Invitrogen) was cultivated in BMGY medium in the phase of growth phase and BMMY medium in the phase of induction. There is 100 mM potassium phosphate (pH 6.0), 1.34% yeast nitrogen base without amino acids, 4 × 10^−5%^ biotin, and 1% glycerol in BMGY medium. And there is 100 mM potassium phosphate (pH 6.0), 1.34% yeast nitrogen base without amino acids, 4 × 10% biotin, and 0.5% glycerol in BMMY medium. Escherichia coli DH5α were used for plasmid propagation while BL-21(DE3) cells were used for cloning of Lac Z coding sequence. Escherichia coli DH5α was cultivated in LB medium with temperature of 37 °C. And LB medium contains 1% tryptone, 0.5% yeast extract and 0.5% NaCl supplemented with 25 µg ml^−1^ Zeocin for plasmid maintenance and propagation.

Plasmids: pPICZA was used for constructing mutants of AOX1 promoter while pGAPZαA (Invitrogen) was used for cloning the sequence of GAP promoter for homologous recombination with yeast genome. All plasmids used in this study are listed in Table 1.

### Construction of promoter mutants and nucleosome positioning prediction

The pGH coding sequence was obtained with the forward primer pGH-F and reverse primer pGH-R being used for clonal expansion of cDNA of porcine pituitary. After digested by EcoR I and Not I, PCR products of pGH were inserted into the vector pPICZA, thus developing pPICZA-pGH. And the expression of pGH was under control of AOX1 promoter. Sequence of GAP promoter was inserted into pPICZA-pGH, producing plasmids which were inserted into GAP promoter locus of genome with homologous recombination. As a result, a homologous region between plasmid and Pichia pastoris was created.

For insert GAP promoter into pPICZA-pGH, complementary chimeric primers Xba-F/Xba-R containing *Xba* I site were used for creating a *Xba* I site upstream of *Bgl*II site on pPICZA-pGH by omega PCR^21^. In omega PCR, the two portions of the chimeric primers annealed to their complementary sites on pPICZA-pGH. The *Xba* I site was to be inserted into the target position of pPICZA-pGH through ~25 cycles of PCR. With processed by *Dpn* I, the PCR product transformed into *E*. *coli* DH5α competent cells. The pPICZA-pGH-Xba transformants were screened by a pair of primers Check-Xba-F/AOX-R.

The GAP promoter fragment was amplified from plasmid pGAPZαA by PCR and digested by *Bgl* II and *Xba* I, and inserted into the vector pPICZA-pGH-Xba resulting in GAP-pPICZA-pGH.

Next, we identified several poly (dA:dT) tracts upstream the basal promoter of *AOX1* (AOX194)^5^ and found three 5 bp perfect poly (dA:dT) tracts, and five AT rich tracts, as shown in Fig. 1. In order to obtain single site deletion variants of *AOX1* promoter, we performed deletion each of these tracts by deletion omega PCR^21^. The primers for deletion of poly (dA:dT) tracts were consist of two parts. The 5′ portion of the forward primer Deletion-S(n)-F(n = 1~8) consisted of 25 bases and was identical to the 5′-flanking sequence of the poly (dA:dT) tracts, and the 3′ portion (~25 bases) was identical to the 3′-flanking sequence of the poly (dA:dT) tracts. Thus, the poly (dA:dT) tract was removed from forward primer. The reverse primer Deletion-S(n)-R was reversed and complementary to the forward primer. In omega PCR, the deletion primers annealed to their complementary sites on wild-type *AOX1* promoter of GAP-pPICZA-pGH. After treatment with *Dpn* I, the PCR product was transformed into *E*. *coli* DH5α competent cells. The transformants GAP-pPICZA-Del-S(n)-pGH were screened by a pair of primers Check(n)-F/AOX-R.

The construction of double sites deletion variants were based on single site deletion variants. Double deletion variant Double-Del-S12 and Double-Del-S24 were obtained by deletion of poly (dA:dT) tracts in S1 and S4 sites on single deletion variant Del-S2 respectively, and the corresponding plasmids were named GAP-pPICZA-Double-Del-S12-pGH and GAP-pPICZA-Double-Del-S24-pGH. We obtained the rest double deletion variants Double-Del-34, Double-Del-46, Double-Del-56, Double-Del-57, Double-Del-58, Double-Del-67 and Double-Del-78 by the same way. The corresponding addition variants (Add-S1~S8 and Double-Add-S12~S78) were created by the same method, except that the primers containing extra 15 bases dA/dTs. A schematic representation of plasmids for variants was shown in Fig. S4.

*Lac Z* coding sequence was amplified from genome of Escherichia coli BL-21(DE3) using primers LacZ-F/LacZ-R. Unfavourable *EcoR* I restriction sites within the *Lac Z* conding sequence was removed using primers LacZ-F/Del-EcoR-R. The PCR product was digested by *EcoR* I and *Not* I, and cloned into pPICZA, resulting in pPICZA-LacZ. The obtained pPICZA-LacZ was digested by *EcoR* I and BamH I,and the fragment containing *Lac Z* was used to substitute *pGH* in plasmids containing promoter variants. All plasmids used in this study were confirmed by DNA sequencing. All primers were listed in Table S1 and recombinant plasmids were listed in Table 1.

When recombinant plasmids were inserted into *GAP* promoter locus of *Pichia pastoris*, the nuclesome positioning and affinity of promoters were predicted by NuPoP software using Hidden Markov model^15^.

### Transformation of *P*. *pastoris* and screening of transformants

*P*. *pastoris* X33 electro-competent cells were transformed with *Bln* I-linearized variants, using an Eppendorf Electroporator 2510 (Eppendorf, Germany) and pulsed at 1.5 KV. After electroporation, 0.5 ml of ice-cold 1 M sorbitol was added immediatedly. The suspension was transferred to a sterile 5 ml tube and added 0.5 ml of YPD (20 g tryptone L^−1^, 10 g yeast extract L^−1^ and 20 g D-glucose L^−1^) followed by incubation 1 h at 30 °C with shaking. After regeneration, aliquots were plated on YPDS (20 g tryptone L^−1^, 10 g yeast extract L^−1^, 20 g D-glucose L^−1^, 1 M sorbitol and 15 g agar L^−1^) plates containing 100 μg/ml Zeocin. Positive transformants were screened by PCR using primers 5′*AOX1*/3′*AOX1*.

### Isolation of genomic DNA from *P*. *pastoris*

Genomic DNA of yeast and Escherichia coli were extracted using Rapid Yeast Genomic DNA Isolation Kit and Bacterial Genomic Isolation Kit (Sangon Biotech China). The quality of genomic DNA was assessed by NanoDrop Spectrophotometer (Thermo Scientific, USA).

### Small-scale expression of reporter gene

Colonies of transformants were cultivated in BMGY or BMMY culture medium containing 100 mM potassium phosphate (pH 6.0), 1.34% yeast nitrogen base without amino acids, 4 × 10–5% biotin, and 1% glycerol (BMGY) or 0.5% methanol (BMMY), respectively. Colonies of transformants were inoculated into 50 ml tube containing 10 ml of BMGY medium with 230 rpm shaking at 28 °C until the optical density reached 15 (OD_600_). The cells were centrifuged at 3,000 g for 10 min at room temperature, and then suspended in 10 ml of BMMY. Methanol was supplied in a final concentration of 0.5% every 24 h. After 48 h of induction, the pellet was collected for subsequent experiments.

### Quantitative real-time PCR assays

The total RNA of yeast and porcine pituitary were extracted using total RNA Isolation Kit (Sangon Biotech China). RNA integrity was checked on 1% agarose gels and quantified using NanoDrop (Thermo Scientific, USA). After heating at 85 °C for 10 mins to denature RNA, 500 ng of total RNA was subjected to reverse transcription using the ReverTra Ace quantitative real-time PCR (qPCR) RT Kit (TOYOBO, Japan). Stationary samples were used for real-time PCR analysis. The level of mRNA was quantified with qPCR using a commercial reagent kit. For each of the targeted genes, a pair of oligonucleotide primers were designed by Primer Premier 5.0 software (As shown in Table S1), based on the sequences registered in GenBank database (GenBank accession number: *actin*: AF216956, *pGH*: x53325, *LacZ:* WP_000177906.1). Values for each target gene were normalized using *actin*. Expression values were calculated using the 2^−△△Ct^ method^22^.

The copy number of *pGH* and *Lac Z* gene in each strain were estimated according to the published method with modifications, *actin* was used as endogenous gene, while *pGH* and *Lac Z* were used as target genes.

### Extraction of intracellular proteins from *P*. *pastoris*

Protein extractions from cytoplasmic and membrane-associated fractions were done according to previous study^23^. Briefly, cells were harvested, 1.5 × 10^8^ cells washed in PBS pH 7.4, and resuspended in 300 ul of yeast breaking buffer. An equal volume of acid-washed glass beads was added and cells were disrupted by vortexing ten times for 1 min with 1-min intervals in ice. The lysate was centrifuged at 10,000 × g for 30 min at 4 °C and supernatant was collected. The pellet was further resuspended in 100ul yeast breaking buffer plus 2% SDS. After centrifugation at 4,000 × g for 5 min at 4 °C, the supernatants containing the membrane-associated proteins were collected. Fifty micrograms of cytoplasmic proteins or membrane-associated proteins determined by BCA protein assay was analyzed by SDS-PAGE and Western blot.

### Western blot assays

Equal amounts of extracted proteins were analyzed on 15% SDS polyacrylamide gel electrophoresis (PAGE). After electrophoresis, the proteins were transferred to PVDF membrane and the membrane was incubated 37 °C with 5% skimmed milk. After incubated with a 1:2000 dilution of Rabbit anti-pGH polyclonal antibody overnight at 4 °C and incubated with AP conjugated goat polyclonal anti-Rabbit IgG (CWBIO China) at a dilution of 1:4000. Immunoreactive bands were visualized with BCIP/NBT Kit (CWBIO China) and estimated by ImageJ software. And nonspecific bands would be shielded, having no influence on quantity the target proteins.

### Beta-galactosidase assay

Cells were harvested. A total of 1.5 × 10^8^ cells were washed in 100 mM Tris-HCl pH 8.0 and re-suspended in 300ul of yeast breaking buffer. An equal volume of acid-washed glass beads was added. Cells were disrupted by vortexing ten times for 1 min with 1 min intervals in ice. The lysate was centrifuged at 10,000 × g for 30 min at 4 °C, and supernatant was collected. Twenty microliters of the supernatant were taken for protein concentration determination using the BCA protein assay. Fifty microliters of the supernatant were then added directly to 0.95 ml of Z buffer to make a total volume of 1 mL. Then 0.2 ml of 4 mg/ml o-nitrophenyl-β-D-galactopyranoside (ONPG) stock solution was added and reactions proceeded as previous method^24^.

## Electronic supplementary material

Supplementary data
