# Supplementary material for: Controlling AOX1 promoter strength in Pichia pastoris by manipulating poly (dA:dT) tracts
Source: Sci Rep. 2018 Jan 23;8:1401. doi: 10.1038/s41598-018-19831-y (PMC5780452; doi:10.1038/s41598-018-19831-y)
Supplement: Supplementary file 1 — Supplementary data [file 41598_2018_19831_MOESM1_ESM.doc]

***Scientific Reports***

**Controlling *AOX1* promoter strength in *Pichia pastoris* by manipulating poly (dA:dT) tracts**

Jun Yang^a,1^, Haiming Cai^a,1^, Jie Liu^a^, Min Zeng^a^, Jiawei Chen^a^, Qingmei Cheng^a^, , Linghua Zhang^*,a^

^a^Guangdong Provincial Key Laboratory of Protein Function and Regulation in Agricultural Organisms, College of Life Sciences, South China Agricultural University, Guangzhou, Guangdong 510642, China

^1^These authors contributed equally to this work.

*Corresponding author. Tel.: 86-13650960875; Fax: 8620-85282180.

E-mail address: [lhzhang@scau.edu.cn](mailto:lhzhang@scau.edu.cn)

**Predicted nucleosome affinity**

a

b

**Predicted nucleosome affinity**

c

**Predicted nucleosome affinity**

d


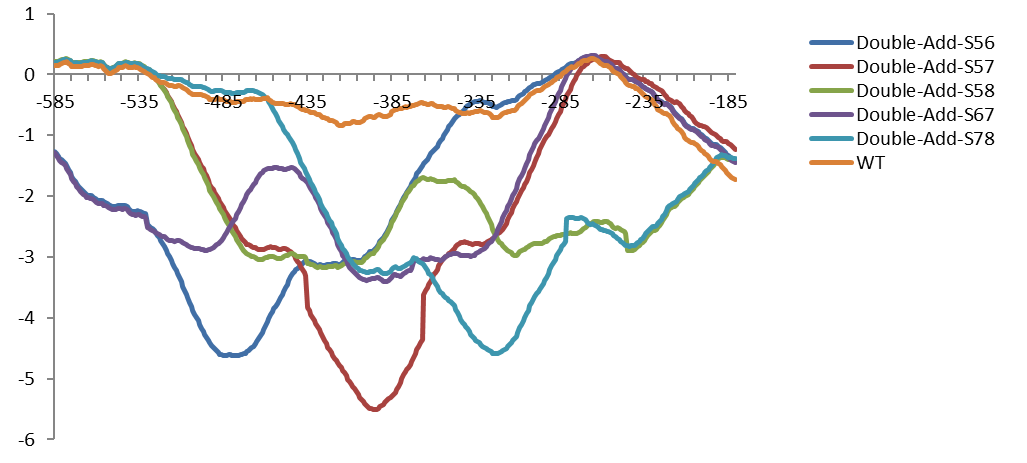


**Predicted nucleosome affinity**

**Figure S1** Predicted Nucleosome affinity of region (-585 to -185) in *AOX1* promoter. Predicted nucleosome affinity profiles generated by NuPoP software for several deletion variants (**a** and **b**) and addition variants (**c** and **d**).

**Predicted nucleosome occupancy**


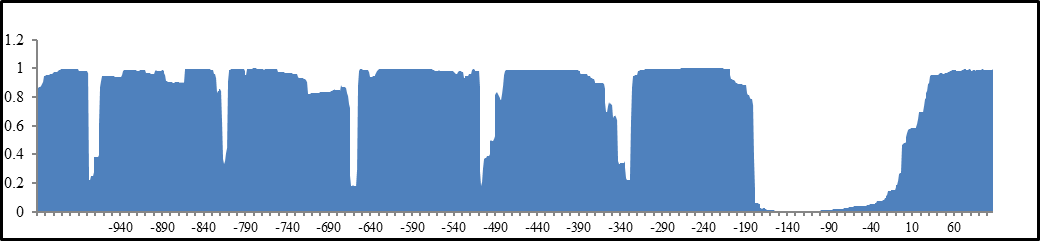


WT

**-5 -4 -3 -2 -1 +1**


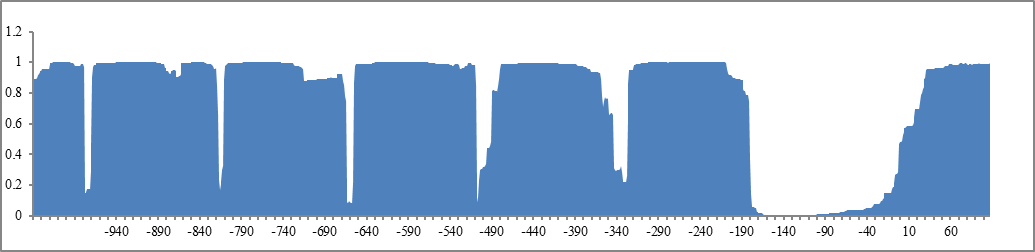


Del-S1

**-5 -4 -3 -2 -1 +1**


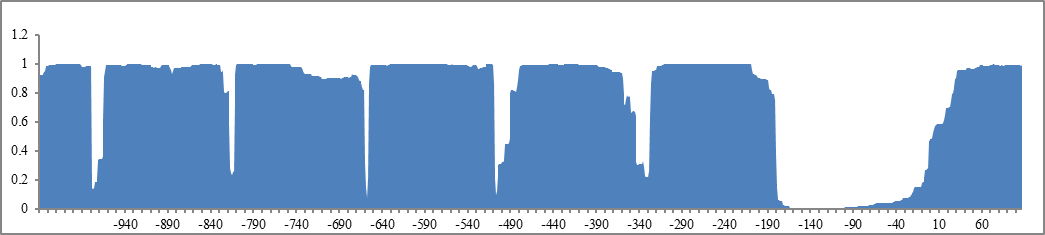


Del-S2

**-5 -4 -3 -2 -1 +1**


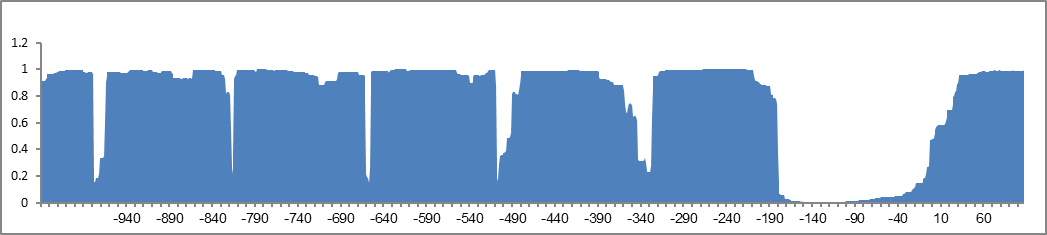


Del-S3

**-5 -4 -3 -2 -1 +1**


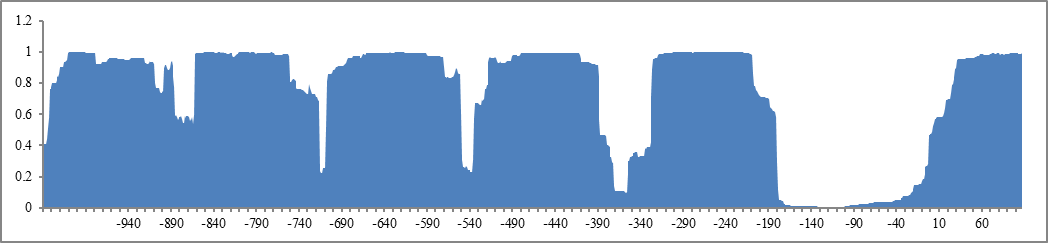


Del-S4

**-5 -4 -3 -2 -1 +1**


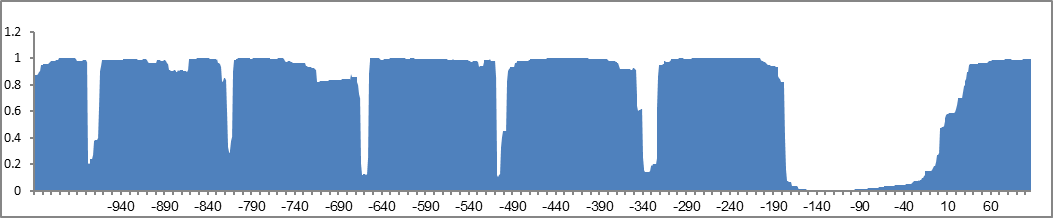


Del-S5

**-5 -4 -3 -2 -1 +1**


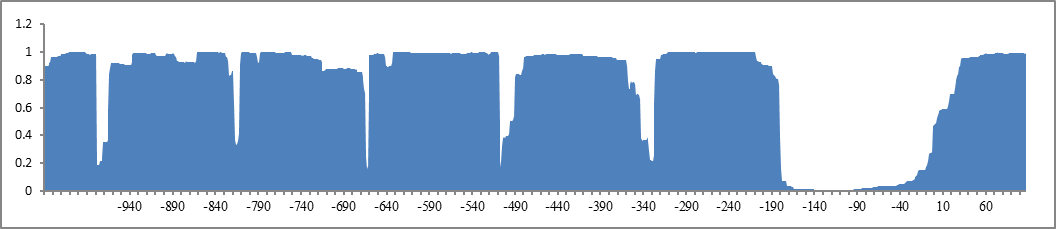


Del-S6

**-5 -4 -3 -2 -1 +1**


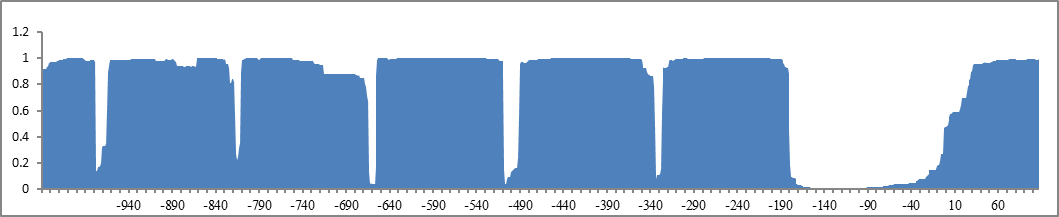


Del-S7

**-5 -4 -3 -2 -1 +1**


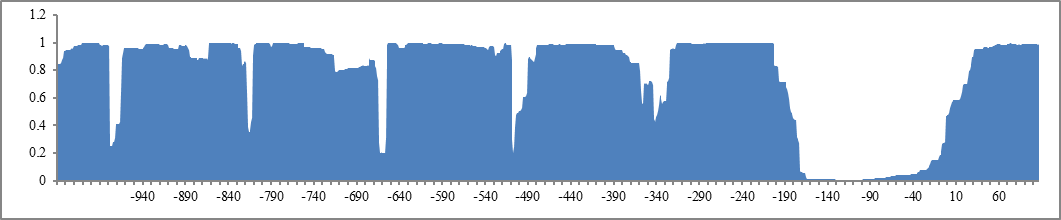


Del-S8

**-5 -4 -3 -2 -1 +1**


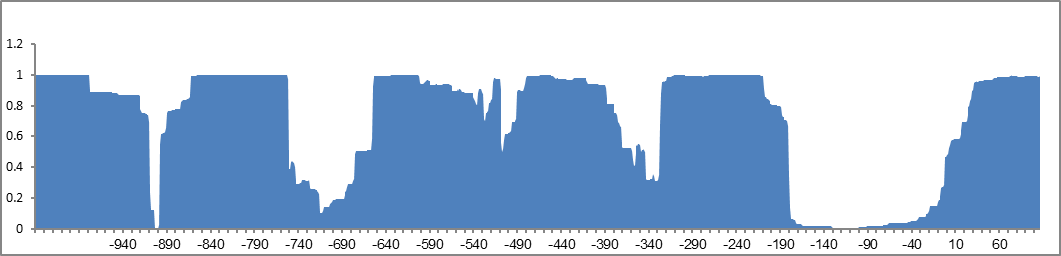


Add-S1

**-5 -4 -3 -2 -1 +1**


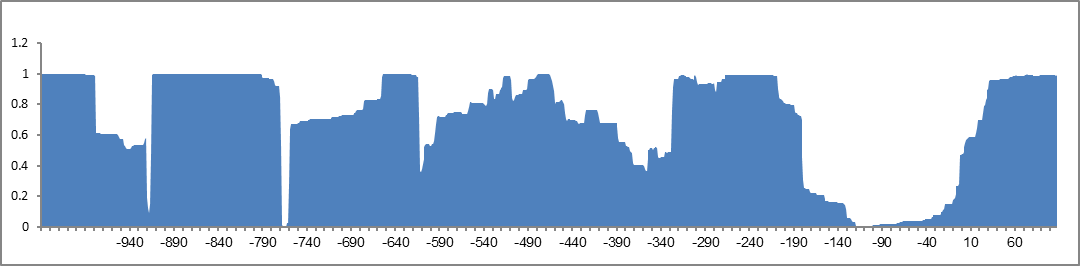


Add-S2

**-5 -4 -3 -2 -1 +1**


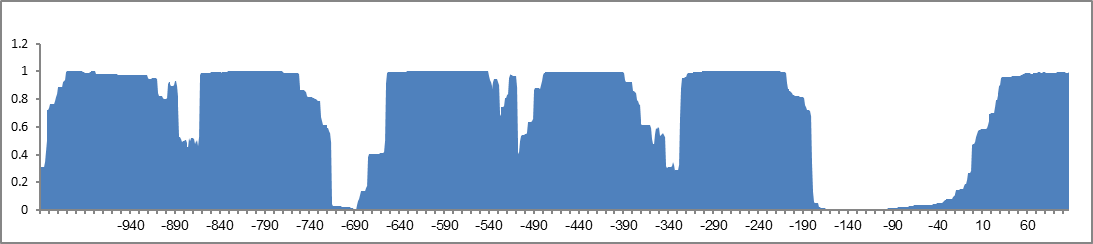


Add-S3

**-5 -4 -3 -2 -1 +1**


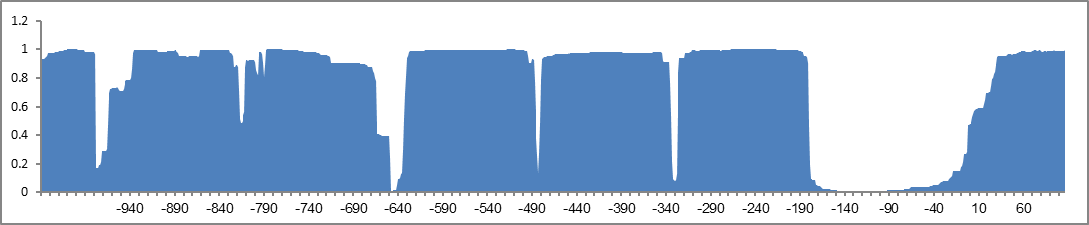


Add-S4

**-5 -4 -3 -2 -1 +1**


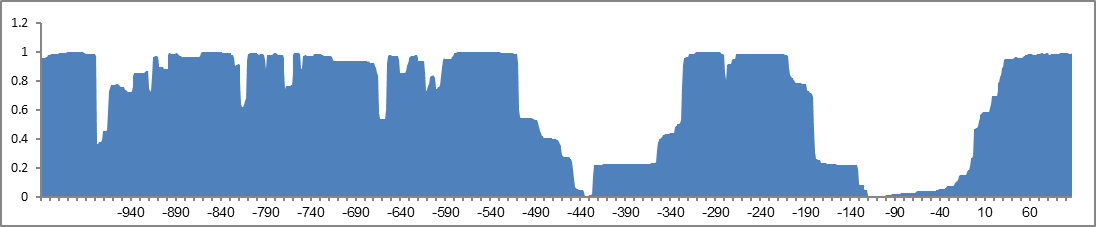


Add-S5

- **-5 -4 -3 -2 -1 +1**


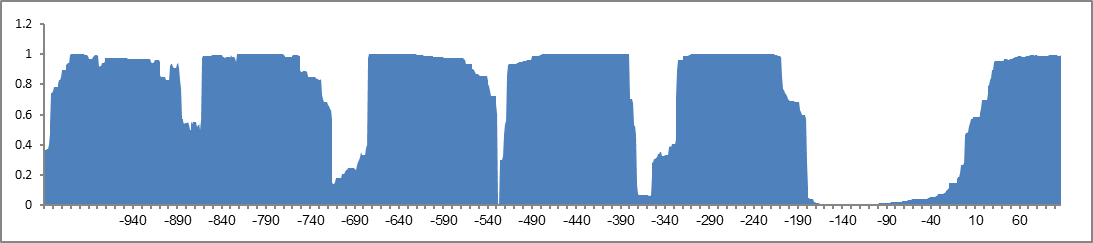


Add-S6

**-5 -4 -3 -2 -1 +1**


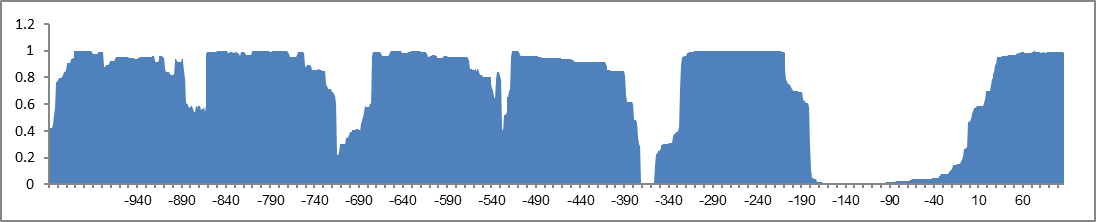


Add-S7

**-5 -4 -3 -2 -1 +1**


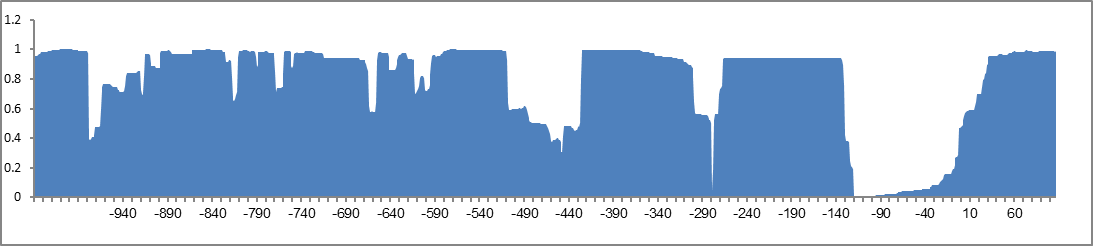


Add-S8

**-5 -4 -3 -2 -1 +1**


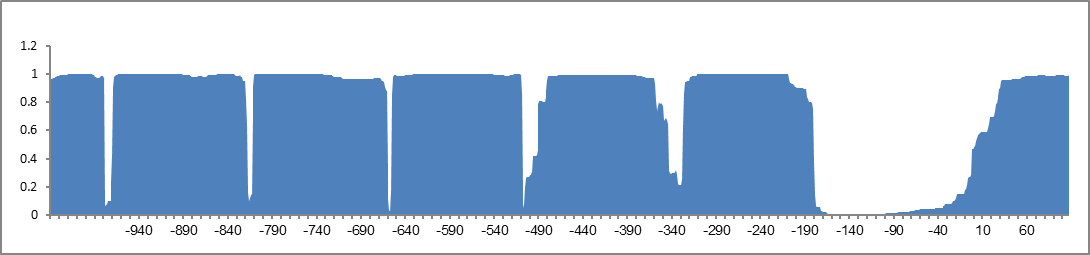


Double-Del-S12

**-5 -4 -3 -2 -1 +1**


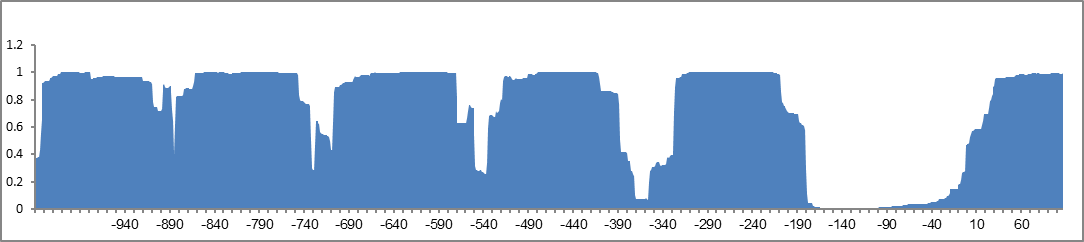


Double-Del-S24

**-5 -4 -3 -2 -1 +1**


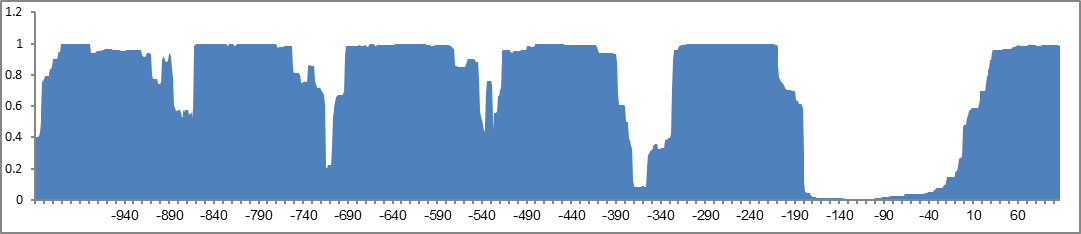


Double-Del-S34

**-5 -4 -3 -2 -1 +1**


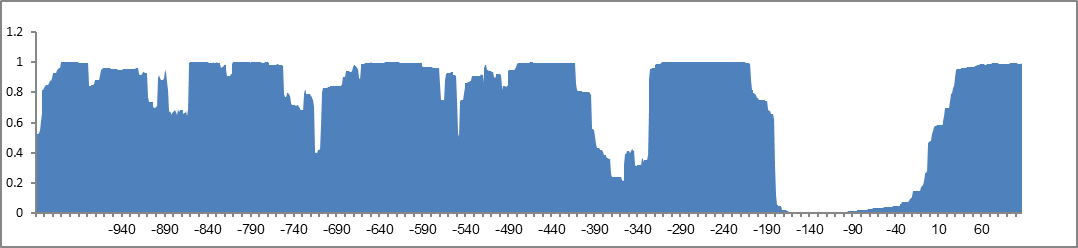


Double-Del-S46

**-5 -4 -3 -2 -1 +1**


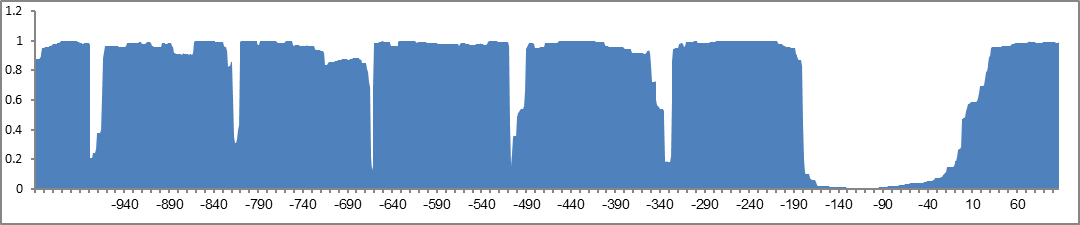


Double-Del-S56

**-5 -4 -3 -2 -1 +1**


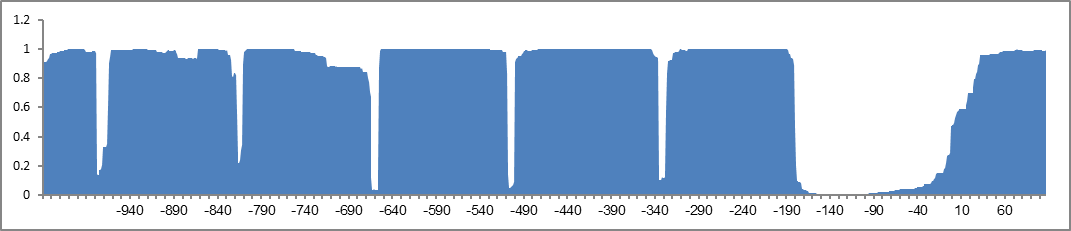


Double-Del-S57

**-5 -4 -3 -2 -1 +1**


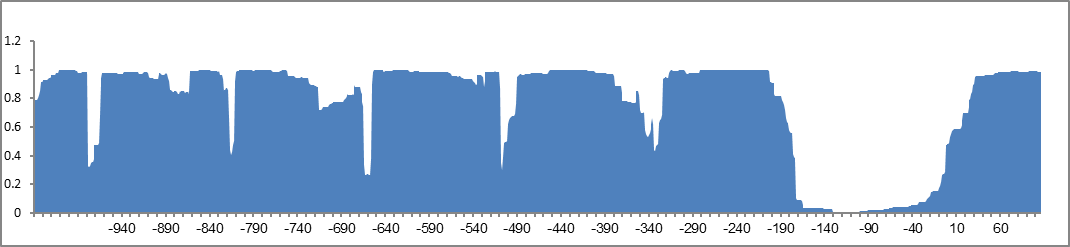


Double-Del-S58

**-5 -4 -3 -2 -1 +1**


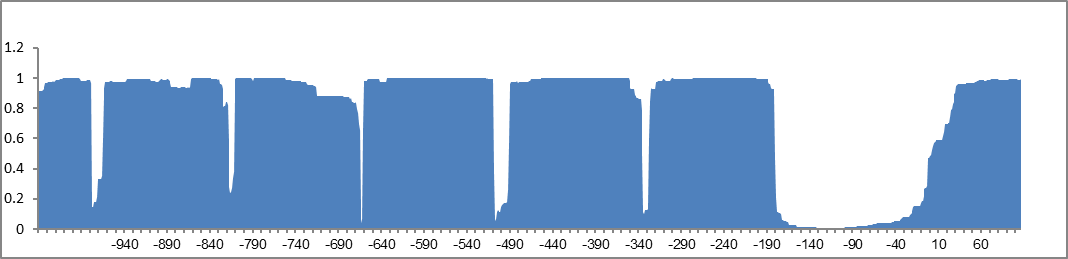


Double-Del-S67

**-5 -4 -3 -2 -1 +1**


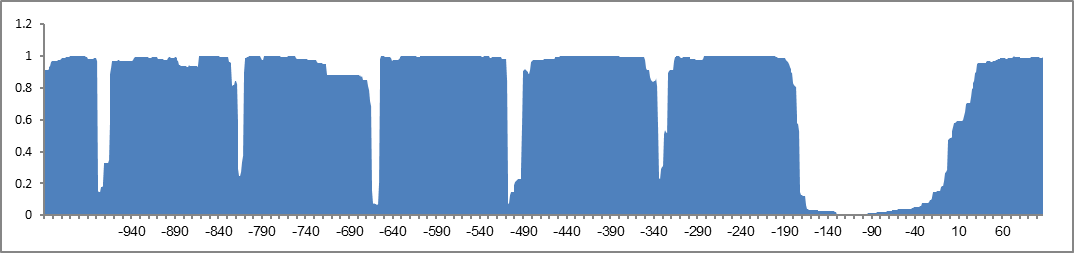


Double-Del-S78

**-5 -4 -3 -2 -1 +1**


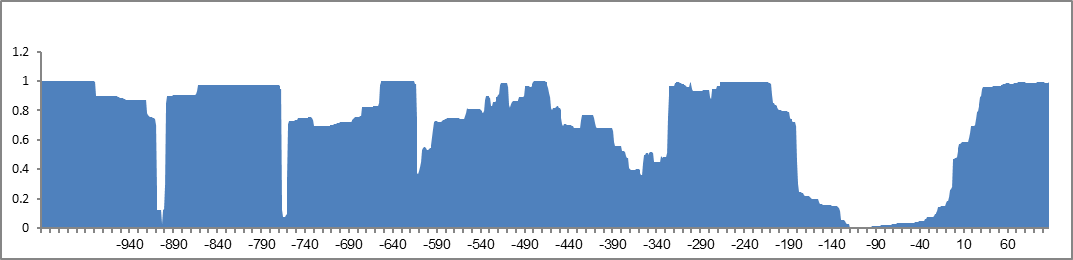


Double-Add-S12

**-5 -4 -3 -2 -1 +1**


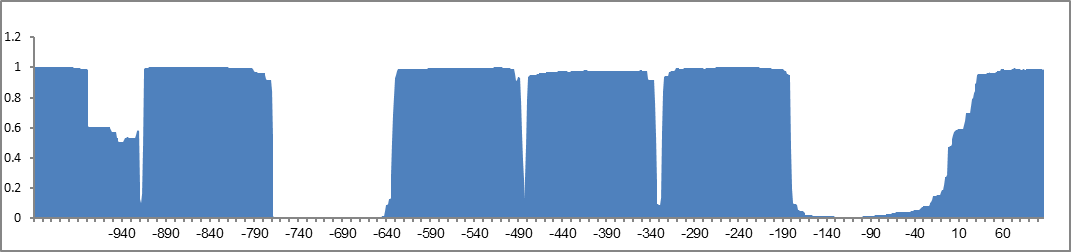


Double-Add-S24

**-5 -4 -3 -2 -1 +1**


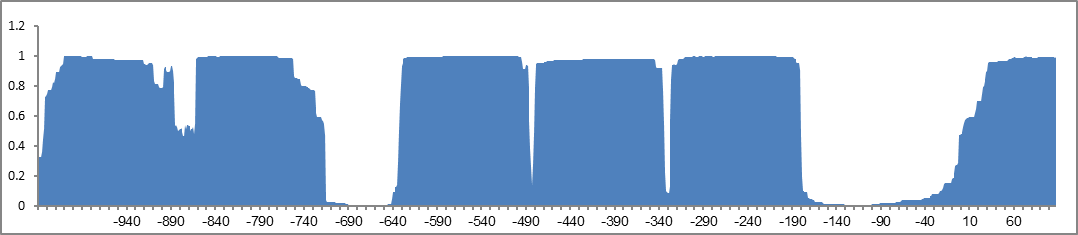


Double-Add-S34

**-5 -4 -3 -2 -1 +1**


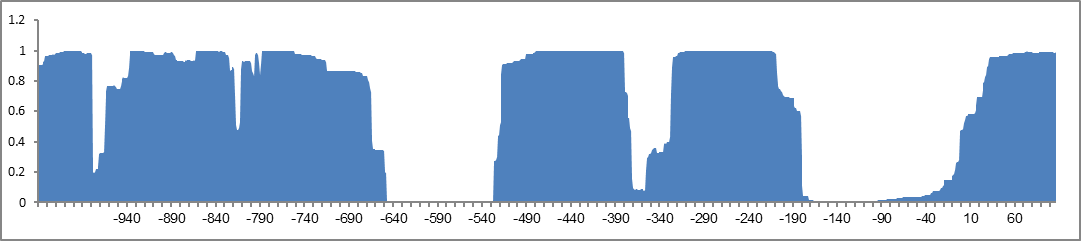


Double-Add-S46

**-5 -4 -3 -2 -1 +1**


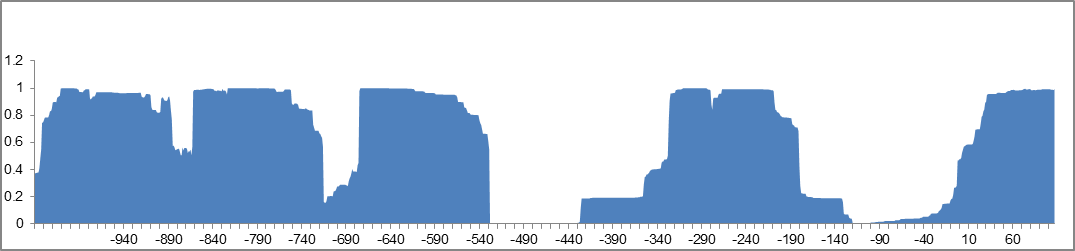


Double-Add-S56

**-5 -4 -3 -2 -1 +1**


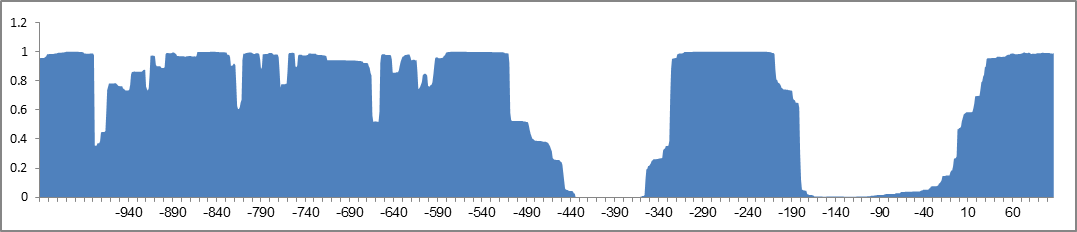


Double-Add-S57

Double-Add-S57

**-5 -4 -3 -2 -1 +1**


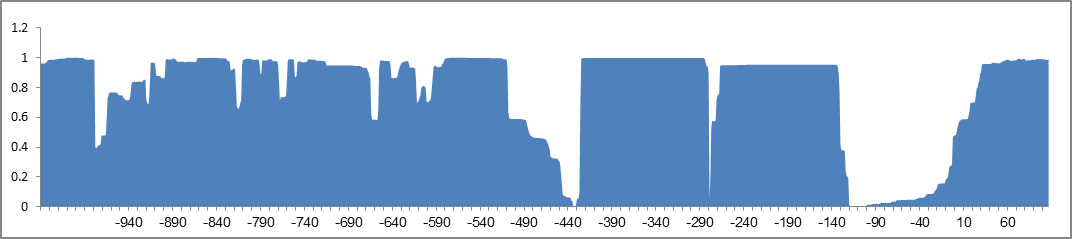


Double-Add-S58

**-5 -4 -3 -2 -1 +1**

Double-Add-S58


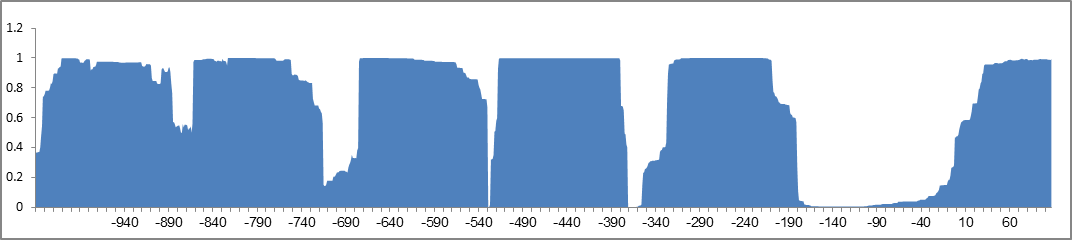


Double-Add-S67

**-5 -4 -3 -2 -1 +1**


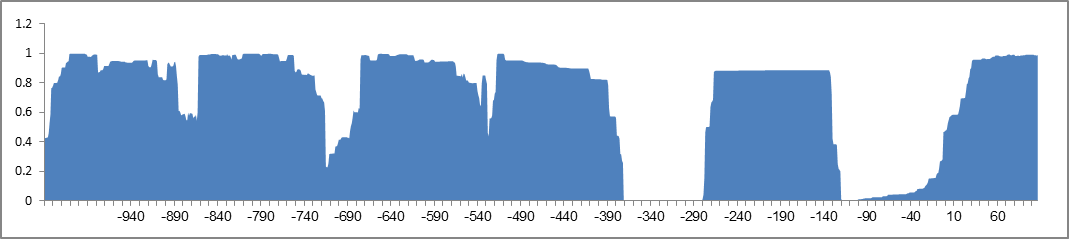


Double-Add-S78

**-5 -4 -3 -2 -1 +1**

**Figure S2** Predicted nucleosome occupancy of AOX1 variants. The nucleosome occupancy of AOX1 promoter by the hidden Markov model which was usually used for predicting genome nucleosome occupancy. Here showed the predicted nucleosome occupancy profiles for all variants which listed in Figure 2.

a

b

c

**Figure S3** Promoter activity in presence of glycerol as represented by beta-galactosidase activity. Beta-galactosidase enzyme activities of variants were compared to wild-type promoter transformant, and are represented by the mean ± SD of three independent cultivations. Relative enzyme activities of deletion variants were shown in a, and relative enzyme activities of addition variants were shown in b. c: Correlation analysis of promoter strength before and after induced by methanol, and correlation coefficient was 0.56.


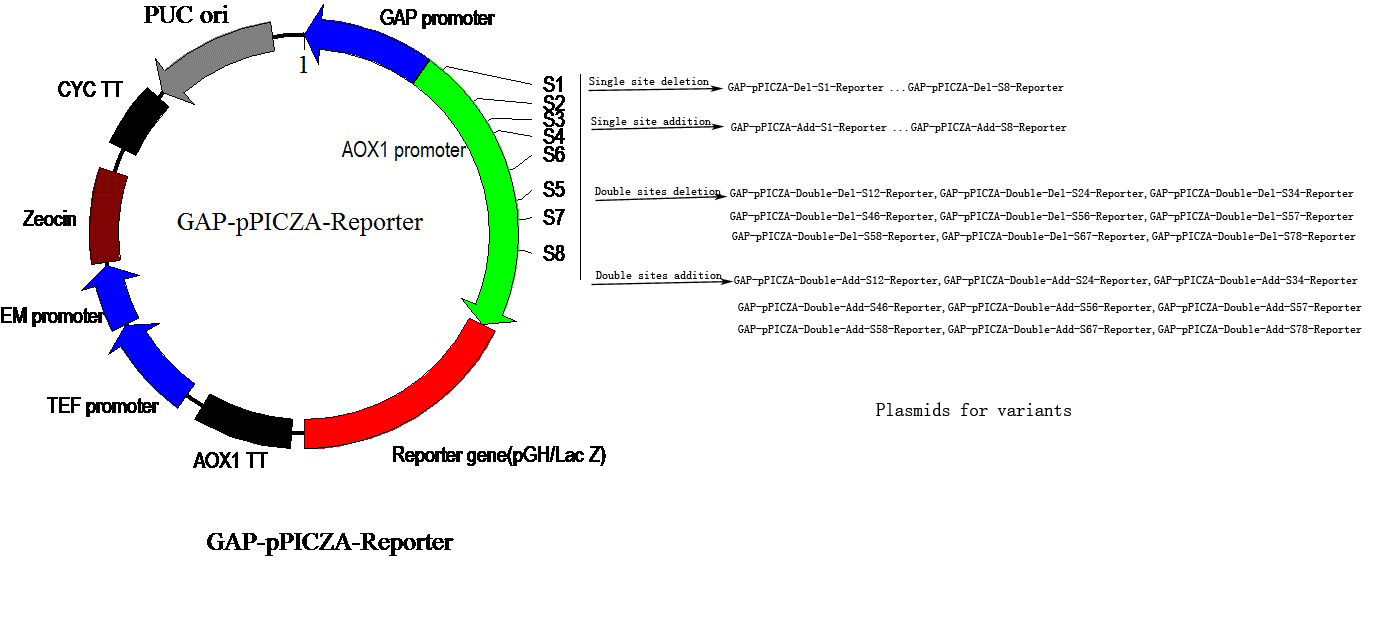


**Figure S4** A schematic representation of plasmids for variants S1~S8 represented the site of poly (dA:dT) tracts; The resulting plasmids were obtained by deletion/addition of poly (dA:dT) tracts.

KDa

20


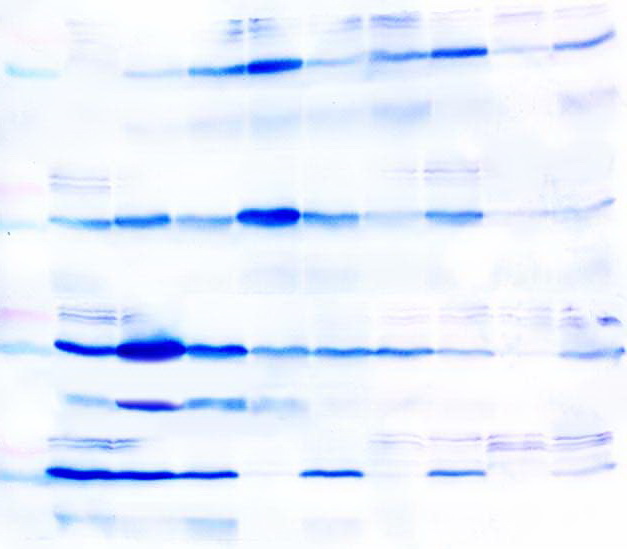


20

20

a

b

C

d

25

25

25

25

20

**Figure S5** The full-length blots for Figure 2**a, b, c** and **d.** Western blots of pGH protein from strains containing PAOX1 variants. For detection of pGH, 50μg intracellular proteins was used for SDS-PAGE and Rabbit anti-pGH polyclonal antibody was used for Western blot. The pGH expression level in poly(dA:dT) tracts deletion variants were presented in Lane 3-9(a) ,Lane 1 (b) and Lane1-9(c); The pGH expression level in addition variants were represented in Lane 2-9(b) and Lane 1-9(d). Here the western blot results are detected with total proteins, not purified pGH protein. So the additional bands are the non-specific reaction of the Rabbit anti-pGH polyclonal antibody. It is no doubt that some bands may larger than the expected size. But the additional bands will be slight and not influence to quantify the target protein pGH.

Table S1 Primers used for reactions

| Primer name | Sequence |
| --- | --- |
| pGH-F | CGGAATTCACCATGTTCCCAGCCATGCCCTTGTCC |
| pGH-R | ATAAGAATGCGGCCGCCTAGAAGGCACAGCTGCTCTCCA |
| Xba-F | ACGAAAACTCTAGAACGTTAAGGGATTTTGGTC |
| Xba-R | ACGTTCTAGAGTTTTCGTTCCACTGAGC |
| Check-Xba-F | CAGTGGAACGAAAACTCTAGA |
| AOX-F | AGATCTAACATCCAAAGACG |
| AOX-R | GTGAATTCCTCGTTTCGAAT |
| GAP-F-Bgl | GAAGATCTTTTTTGTAGAAATGT |
| GAP-R-Xba | GCTCTAGACGTTTCGAAATAGTTGTTCAA |
| Lac-F | CGGAATTCACCATGACCATGATTACGGATTCAC |
| Lac-R | ATAAGAATGCGGCCGCTTATTTTTGACACCAGACCAACT |
| Del-EcoR-R | TTATTTTTGACACCAGACCAACTGGTAATGGTAGCGACCGGCGCTCAGCTGAAATTCCG |
| 5’AOX1 | GACTGGTTCCAATTGACAAGC |
| 3’AOX1 | GCAAATGGCATTCTGACATCC |
| Deletion-S1-F | TTGAATGAAACCGCCATCCGACATCCACAGGTCCA |
| Deletion-S1-R | GGACCTGTGGATGTCGGATGGCGGTTTCATTCAAC |
| Check 1-F | AGGTTGAATGAAACCGCC |
| Deletion-S2-F | CCTCAACACCCACTTTTGCCATCGCCAGCCCAGTTATTGG |
| Deletion-S2-R | CCAATAACTGGGCTGGCGATGGCAAAAGTGGGTGTTGAGG |
| Check 2-F | CACCCACTTTTGCCATCGCCAG |
| Deletion-S3-F | TTAGGCTACTAACACCATGACGCCTGTCTATCCTG |
| Deletion-S3-R | CAGGATAGACAGGCGTCATGGTGTTAGTAGCCTAA |
| Check 3-F | TTAGGCTACTAACACCATGACGCC |
| Deletion-S4-F | CCCCCCTGGCGAGGTTCATGCCGAATGCAACAAGCTCCG |
| Deletion-S4-R | ATGCGGAGCTTGTTGCATTCGGCATGAACCTCGCCAG |
| Check 4-F | CCCCCCTGGCGAGGTTCATGCCG |
| Deletion-S5-F | ATGCTAACGGCCAGTTGGTCCTTCCAAAAGTCGG |
| Deletion-S5-R | ACTTTTGGAAGGACCAACTGGCCGTTAGCAT |
| Check 5-F | ATGCTAACGGCCAGTTGGTCCTTCC |
| Deletion-S6-F | TGGCCCAAAACTGACAGCGCTGTCTTGGAACC |
| Deletion-S6-R | GGTTCCAAGACAGCGCTGTCAGTTTTGGGCCA |
| Check 6-F | TGGCCCAAAACTGACAGCGC |
| Deletion-S7-F | TGGTATTGATTGACGAATGCTCTCTCATTAATGCTTAGCGCAGTCT |
| Deletion-S7-R | ACTGCGCTAAGCATTAATGAGAGAGCATTCGTCAATCAATACCA |
| Check 7-F | TGGTATTGATTGACGAATGCTCTCT |
| Deletion-S8-F | TGGGGAAACACCCGCGGATGATTATGCATTGTCT |
| Deletion-S8-R | AGACAATGCATAATCATCCGCGGGTGTTTCCCCA |
| Check 8-F | ATGGGGAAACACCCGCGGA |
| Addition-S1-F | TTGAATGAAACCTTTTTTTTTTTTTTTTTTTTGCCATCCGACATCCACAGGTCCA |
| Addition-S1-R | GGACCTGTGGATGTCGGATGGCAAAAAAAAAAAAAAAAAAAAGGTTTCATTCAAC |
| Addition-S2-F | CTCAACACCCACTTTTGCCATCGAAAAAAAAAAAAAAAAAAAACCAGCCCAGTTATTGG |
| Addition-S2-R | CCAATAACTGGGCTGGTTTTTTTTTTTTTTTTTTTTCGATGGCAAAAGTGGGTGTTGAG |
| Addition-S3-F | TTAGGCTACTAACACCATGACTTTTTTTTTTTTTTTTTTATTAGCCTGTCTATCCTG |
| Addition-S3-R | CAGGATAGACAGGCTAATAAAAAAAAAAAAAAAAAAGTCATGGTGTTAGTAGCCTAA |
| Addition-S4-R | CTGGCGAGGTTCATGTTTTTTTTTTTTTTTTTTGTTTATTTCCGAATGCAACAAGCTCC |
| Addition-S4-R | GGAGCTTGTTGCATTCGGAAATAAACAAAAAAAAAAAAAAAAAACATGAACCTCGCCAG |
| Addition-S5-F | ATGCTAACGGCCAGTTGGTCAAAAAAAAAAAAAAAAAAAAGAAACTTCCAAAAGTCGG |
| Addition-S5-R | ACTTTTGGAAGTTTCTTTTTTTTTTTTTTTTTTTTGACCAACTGGCCGTTAGCAT |
| Addition-S6-F | TGGCCCAAAACTGACAGTTTTTTTTTTTTTTTTTTAAACGCTGTCTTGGAACC |
| Addition-S6-R | GGTTCCAAGACAGCGTTTAAAAAAAAAAAAAAAAAACTGTCAGTTTTGGGCCA |
| Addition-S7-F | TTGATTGACGAATGCTCAAAAAAAAAAAAAAAAAAAATAATCTCATTAATGCTTAGCGC |
| Addition-S7-R | GCTAAGCATTAATGAGATTATTTTTTTTTTTTTTTTTTTTGAGCATTCGTCAATCAATA |
| Addition-S8-F | TGGGGAAACACCCGCTTTTTTTTTTTTTTTTTTTTGGATGATTATGCATTGTCT |
| Addition-S8-R | AGACAATGCATAATCATCCAAAAAAAAAAAAAAAAAAAAGCGGGTGTTTCCCCA |
| AOX-F(qPCR) | TCCACAGGTCCATTCTCACACA |
| AOX-R(qPCR) | GCTCCAATCAAGCCCAATAACT |
| Actin-F(qPCR) | CCTGAGGCTTTGTTCCACCCATCT |
| Actin-R(qPCR) | GGAACATAGTAGTACCACCGGACATAACGA |
| pGH-F(qPCR) | AGAGGTACTCCATCCAGAAC |
| PGH-R(qPCR) | CAGGCTGTTGGTGAAGAC |
